# Using short-term endpoints to improve interim decision making and trial duration in two-stage phase II trials with nested binary endpoints

**DOI:** 10.1177/09622802231188515

**Published:** 2023-07-25

**Authors:** Dario Zocholl, Cornelia U. Kunz, Geraldine Rauch

**Affiliations:** 1 Charité - Universitätsmedizin Berlin, Corporate member of Freie Universität Berlin and Humboldt-Universität zu Berlin, Institute of Biometry and Clinical Epidemiology, Berlin, Germany; 2 Biostatistics and Data Sciences, Boehringer Ingelheim Pharma GmbH & Co. KG, Biberach/Riss, Germany

**Keywords:** Adaptive clinical trials, conditional power, posterior predictive probability of success

## Abstract

In oncology, phase II clinical trials are often planned as single-arm two-stage designs with a binary endpoint, for example, progression-free survival after 12 months, and the option to stop for futility after the first stage. Simon’s two-stage design is a very popular approach but depending on the follow-up time required to measure the patients’ outcomes the trial may have to be paused undesirably long. To shorten this forced interruption, it was proposed to use a short-term endpoint for the interim decision, such as progression-free survival after 3 months. We show that if the assumptions for the short-term endpoint are misspecified, the decision-making in the interim can be misleading, resulting in a great loss of statistical power. For the setting of a binary endpoint with nested measurements, such as progression-free survival, we propose two approaches that utilize all available short-term and long-term assessments of the endpoint to guide the interim decision. One approach is based on conditional power and the other is based on Bayesian posterior predictive probability of success. In extensive simulations, we show that both methods perform similarly, when appropriately calibrated, and can greatly improve power compared to the existing approach in settings with slow patient recruitment. Software code to implement the methods is made publicly available.

## 1. Introduction

In oncology, phase II clinical trials are often carried out to test in a specific population of patients whether a therapy is sufficiently effective to warrant further investigation. Due to both ethical and practical constraints, sample sizes of such trials are typically strictly limited. Therefore, many trials apply a single-arm design to explore the treatment’s efficacy before initiating a larger randomized phase II or III clinical trial. Endpoints in such single-arm trials are usually binary, such as response according to Response Evaluation Criteria In Solid Tumors criteria or rate of progression-free survival (PFS) at a specific time point. PFS itself is naturally a time-to-event endpoint, but the measurements of progression are often not continuous but occur in a few discrete time steps, for example, every 3 months. In many trials an option to stop for futility after an interim analysis is implemented. A popular design that allows stopping for futility for a single-arm trial with a binary endpoint is Simon’s two-stage design.^1^ This design requires at least a fixed number of responses/survivors being observed in the patients of the first stage to allow continuation to the second stage. The popularity of the design can be accounted to a large extent to the simplicity of the design as well as its relatively small sample size, which often allows conducting of the studies in a single center, thus reducing the expenses of the trial.

If the primary endpoint is assessed sometime after recruitment or treatment initiation, for example, if the endpoint is PFS after 12 months confirmed via magnetic resonance imaging, it may happen that there is a severe delay due to the interim analysis, since the second stage usually cannot be initiated before a sufficient number of survivors was observed in the first stage. This enforced trial interruption not only prolongs drug development, but might also negatively affect the momentum and discourage involved partners. In such situations, it would be attractive to use information on short-term endpoints to guide the decision making whether to stop for futility or to continue, so that there is little or no delay during the trial conduct. As a solution to this problem, Kunz et al.^2^ proposed a method that uses a short-term endpoint, for example, 3-month PFS, for the interim decision, but for the final decision at the end of the trial, a more relevant long-term endpoint, for example, 12-month PFS, is used as a primary endpoint. The approach requires making explicit a priori assumptions about the short-term endpoint, and therefore about its correlation with the long-term endpoint. It is possible to specify this assumption in the form of weakly informative distributions, but still, a fixed threshold of short-term survivors that need to be observed to make the interim decision is set before patient recruitment is initialized.

A similar approach to Kunz et al.^2^ was presented recently by deVeaux et al.^3^ Here, also a fixed threshold for the number of short-term responders is set and particular emphasis is put on the trial duration, which depends on the patient accrual rate. Within the Bayesian framework, Lin et al.^4^ proposed a Bayesian time-to-event method to evaluate futility by taking into account the time that patients with pending outcomes have already spent in the trial in single-arm multi-stage designs. However, the method cannot handle early outcome assessments, and once again fixed thresholds for the interim decision are set in the planning stage.

When we planned an actual phase II clinical trial in the field of glioblastoma research and considered implementing the design by Kunz et al., the clinicians were very hesitant to provide any assumptions about the short-term endpoint, which would determine the threshold for stopping for futility and hence the fundamental design of the clinical trial. Even the assumption about the long-term endpoint 12-months PFS was a rather rough estimate based on a single study in similar patients, but for any earlier assessments of PFS, not even historical data was available because published studies only reported data for 12-months PFS. This was our motivation to seek a design that allows incorporation of short-term endpoints into the decision without having to make additional assumptions.

A concept that is naturally suited to guide interim decisions in clinical trials such as stopping for futility, is conditional power (CP), which is the statistical power at a time of some interim analysis given the already observed data.^5^ In the randomized clinical trial setting, recent studies used the concept of CP to evaluate futility,^6,7^ and proposed to further improve the decision-making process by adding information from prognostic baseline covariates.^8^ A similar concept to CP is the Bayesian posterior predictive probability of success (PoS).^9,10^ The PoS evaluates the current probability of reaching the trial’s final goal under consideration of all currently available information. It can be used regardless of whether the primary analysis is Bayesian or frequentist.^10,11^ There have been approaches to combine the PoS and multi-stage designs similar to Simon’s design.^12,13^ For time-to-event endpoints, Waleed et al.^14^ proposed a method to evaluate futility using the Bayesian PoS and allows more than one interim analysis. However, this method assumes continuous assessment of the endpoints and requires relatively large sample sizes.

Using both the CP and the PoS framework, we propose two new methods for futility stopping in single-arm phase II oncology settings: STE–CP and STE–PoS (combining **s**hort-**t**erm **e**ndpoints and the **CP** or the **PoS**, respectively). The two methods allow stopping for futility based on early assessments of a discrete-time survival endpoint and do not require any explicit prior assumptions about the early assessments, particularly no assumptions about their correlation with the long-term endpoint or the underlying survival function. It is assumed that the endpoint assessments occur at discrete time points and that assessments are nested, that is, only patients with short-term survival can be long-term survivors. At the end of the trial, the null hypothesis is rejected based on the observed number of survivors on the long-term endpoint, just as in most single-arm two-stage designs. We show how the design parameters can be calibrated such that frequentist operating characteristics under null or alternative hypothesis are approximately fulfilled.

In Section 2, we present an overview of the existing approaches on which the new methods are built upon. In Section 3, we introduce the new methods STE–CP and STE–PoS. In Section 4, we present a simulation study to assess the operating characteristics of the new methods and compare them to the existing designs, considering various scenarios for the true underlying survival functions as well as different patient accrual rates. We conclude with a discussion in Section 5.

## 2. Methods

### 2.1. Simon’s two-stage design

We consider the case of a standard phase II single-arm clinical trial in the field of oncology with a binary endpoint with the option to stop for futility. Such a design was proposed by Simon^1^ and is widely applied today. The original work was using response as an endpoint, but we will focus on its application to survival endpoints with discrete time-to-event measurements, which is a common situation in clinical trials, for example, when tumor progression is assessed via magnetic resonance imaging every 3 months. Simon’s two-stage design tests the hypothesis $H_{0}:p\leq p_{0}$ versus $H_{1}:p\geq p_{1}$ after the second stage. Here, $p_{0}$ denotes the expected survival rate at a specific time point with the standard treatment, and $p_{1}$ represents the desired survival rate of a clinically relevant improvement. In the first stage, $n_{\text{I}}$ patients are recruited, and if there are more than $r_{1}$ survivors observed, the trial continues to the second stage to recruit another $n_{\text{II}}$ patients. If among these $n_{I}+n_{II}=n$ patients more than r survivors are observed, the null hypothesis is rejected and the trial claims success. The design parameters $n_{I},n,r_{1},$ and r are calibrated under a predefined maximal sample size $n_{\text{max}}$ such that the pre-specified type I error rate and power are met. The design that minimizes the expected sample size under the null hypothesis is called the “optimal design” and the design with the smallest total sample size is called the “minimax design”.

For futility stopping, an important operating characteristic of Simon’s two-stage design is the probability of early termination (PET). Under the null hypothesis, the PET should be large, while under the alternative hypothesis, it should be small, and definitely not larger than 1−power . For Simon’s design, the PET can be calculated as

$${\text{PET}}_{\text{Simon}}=B(r_{1};p,n_{I})$$

where B denotes the cumulative binomial distribution.

### 2.2. Two-stage trials using a short-term endpoint

In a two-stage trial, such as Simon’s two-stage design, recruitment of patients is usually stopped after the first stage until enough survivors have been observed among the $n_{\text{I}}$ patients of the first stage. One approach to shorten this forced interruption in the interim was proposed by Kunz et al.^2^ In their paper, they proposed to use a short-term endpoint (“intermediate endpoint” in the original publication) to make an interim decision about trial continuation, while the primary endpoint for the final conclusion at the end of the trial is a long-term endpoint. While the application of this approach is not limited to nested endpoints, we will consider only this situation, particularly survival endpoints.

The hypothesis tested at the end of the trial is the same as in Simon’s two-stage design, that is, $H_{0}:p_{\text{LTE}}\leq p_{\text{LTE},0}$ versus $H_{1}:p_{\text{LTE}}\geq p_{\text{LTE},1}$ , where $p_{\text{LTE}}$ denotes the survival rate of the long-term endpoint. However, after the first stage, the trial only continues if there are more than $s_{1}$ survivors on the short-term endpoint. The design parameters $n_{I},n,s_{1},$ and r are calibrated under the constraints on type I error rate and power, so additionally the survival rates $p_{\text{STE},0}$ and $p_{\text{STE},1}$ of the short-term endpoint under the null hypothesis and the alternative hypothesis need to be specified. Just as with Simon’s design, the minimax and optimal design can be found by an extensive search under the restriction of a maximal sample size $n_{\text{max}}$ . Formulas to calculate power and type I error rate are provided by Kunz et al.^2^ To avoid excessive type I error rates in case of misspecification of $p_{\text{STE},0}$ , Kunz et al. define the design such that even if $p_{\text{STE},0}=1$ , that is, the trial never stops for futility, and therefore the type I error rate is strictly controlled. This is called a non-binding stopping rule. However, to calculate the power under the alternative hypothesis, a binding stopping rule is assumed, hence the short-term rate does affect the power. We define the PET independent from the considered hypothesis as:

$${\text{PET}}_{\text{Kunz}}=B(s_{1};p_{\text{STE}},n_{I})$$

If the true $p_{\text{STE}}$ is smaller than anticipated under the alternative hypothesis, ${\text{PET}}_{\text{Kunz}}$ will be inflated even though the alternative hypothesis for the long-term endpoint may actually be correct. We argue that by using a fixed number of short-term survivors as a threshold for futility stopping, there is a strong requirement for accurate assumptions about the underlying time-to-event curve, which may be difficult when planning a phase II trial. In the setting of early phase clinical trials, often there is limited knowledge about the expected outcomes and even the assumptions on the long-term endpoint are rather rough guesses. Data for other endpoints than the established long-term endpoint is scarce, if even existing, and therefore any assumptions made during the planning phase may not be reliable. We therefore aimed to develop a method that uses the interim data to guide decision making in a two-stage trial while making as few assumptions as possible about this interim data. For this, we applied the frameworks of CP and PoS, which are introduced in the following.

### 2.3. Approaches to interim monitoring

At the time point of interim analysis in a phase II clinical trial, the key question that is addressed is: Is the trial sufficiently likely to achieve its goal based on the data observed so far, or should the trial be stopped for futility? There exist two frameworks that are both naturally suited to answer this question: the CP^15,5^ and the PoS.^10,9,16^ In the literature, the terminology is not entirely consistent, so in the following their use in this manuscript is defined. Generally speaking, both approaches are used to determine the probability of trial success, that is, rejecting the null hypothesis at the end of the study, given the data observed so far. They differ in how they incorporate the information about the data observed so far. The CP is determined based on the assumption about an unknown parameter θ . Multiple ways exist to specify this assumption, for example, using the value that was defined under the alternative hypothesis at the design stage of the trial, but in the following, we only consider using the estimator $\hat{\theta }$ , which is derived from the observed data D . Based on this estimator, future data is predicted, from which the probability of observing more successes x than the critical threshold r and thus rejecting the null hypothesis at the end of the trial, that is, $Pr(x>r|\hat{\theta })$ , can then be calculated. For PoS, the unknown parameter θ is expressed as its posterior distribution given the prior and the observed data, $P_{\text{post}}(\theta )=P(\theta |P_{\text{prior}}(\theta ),\text{D})$ , which is used to calculate the probability of trial success averaged over the posterior distribution, that is, $Pr(x>r|P_{\text{post}}(\theta ))$ . Therefore, in contrast to the CP, the PoS considers also the variability of the parameter based on the current data.

## 3. New approaches: STE–CP and STE–PoS

### 3.1. Motivation

If recruitment is rather slow, as in many single-arm phase II trials, it is likely the interim data contains information not only on the short-term assessments of the endpoint but also on later assessments or even the long-term outcome, either because some patients already had the event and hence cannot be event-free at the long-term endpoint or because they have been observed for so long that their long-term outcome is known. This information cannot be used in Kunz’s design for the interim decision. In contrast, our new methods incorporate this information to evaluate the CP or the PoS in the interim analysis, while still allowing for the same reduction of delay (compared to Simon’s design) due to the interim analysis as Kunz’s design. The trial will continue only if the CP or the PoS are sufficiently large in an interim analysis, which can be conducted at any (prespecified) point of time during the trial.

### 3.2. General notation

In the following, we will introduce the notation used by us. A summary is provided in Table 1. The term “survival” will be used to refer to time-to-event endpoints in general, including particularly PFS, and the term “time point” to refer to the predefined time of the measurement or assessment of a patient’s survival status. We assume that a patient’s survival is measured at discrete time points k∈{0,…,K} , where the outcome of a measurement is either survival or death and k=0 indicates the baseline of recruitment, at which each patient is alive. The outcomes are nested, that is, if a patient is dead at time point k−1 , the patient is known to be dead and cannot be measured at time point k . The trial is supposed to test the probability of survival at the long-term time point $k^{*}$ against some specific constant, so the null hypothesis is $H_{0}\;:p_{k^{*}}\leq p_{k^{*}\;,\;0}$ . For simplicity, we will in the following consider the situation $k^{*}=K$ , that is, the long-term endpoint is also the last measurement time point. The number of patients, who have been measured at time point k is denoted by $n_{k}$ , among which there are $x_{k}$ survivors. At each time point k , the number of survivors follows a binomial distribution, $x_{k}∼\text{Binom}(n_{k},p_{k|k-1})$ , where $p_{k|k-1}$ is the probability of survival at time point k conditional on survival at time point k−1 . Provided the data D , consisting of $x_{k}$ and $n_{k}$ for each $k\in \{0,\ldots ,k^{*}\}$ , the likelihood function for the conditional probability of survival at $k^{*}$ given survival at a previous time point $k^{\prime }<k^{*}$ is:

(1) $$L\left(\left(p_{k^{\prime }+1|k^{\prime }}\right),\ldots ,\left(p_{k^{*}|k^{*}-1}\right)|D\right)=\prod_{k=k^{\prime }+1}^{k^{*}}{\left(p_{k|k-1}\right)}^{x_{k}}{\left(1-p_{k|k-1}\right)}^{n_{k}-x_{k}}$$

which is the product of the probability functions of the binomial distributions of observations $x_{k}\text{ for }k\in \{k^{\prime }+1,\ldots ,k^{*}\}$ . From this, the maximum likelihood estimator of $p_{k^{*}|k^{\prime }}$ arises as the Kaplan–Meier estimator^17^:

(2) $${\hat{p}}_{k^{*}|k^{\prime }}=\left\{ \begin{array}{ll} \prod_{k=k^{\prime }+1}^{k^{*}}\frac{x_{k}}{n_{k}}=\prod_{k=k^{\prime }+1}^{k^{*}}{\hat{p}}_{k|k-1}, & \text{if }k^{\prime }<k^{*}\\ 1, & \text{if }k^{\prime }=k^{*} \end{array}\right.$$

In typical survival analysis at the end of the trial only the probability of survival after being recruited, that is, with the common baseline $k^{\prime }=0$ , is of interest. However, during the interim analysis, the question is: what is the probability that there will be enough long-term survivors at the end of the trial to successfully reject the null hypothesis? To answer this question, we will need to consider that each patient may have a different probability to be a long-term survivor depending on their current survival time. How these current survival times can be incorporated to improve the interim decision, will be shown in the following subsections.

A key concept of survival analysis is censoring. Since in early phase II trials the time-to-event outcomes are typically treated as binary endpoints with primary evaluation at a specific time point, for which censoring technically does not exist, censoring due to loss to follow-up is commonly treated as failure in order to follow a conservative analysis strategy. Typically, this is expected to happen infrequently, because these trials are very small and follow-up is rather short. However, if the interim analysis is performed before all recruited patients have had their long-term outcome observed, there is administrative censoring during the interim. Patients who have been censored in this way, are taken into account in equations (1) and (2) and will be considered accordingly in the decision process as explained hereinafter.

**Table 1.** Glossary of terms.

Term	Description
CP	Conditional power
STE–CP	Method for interim decision making based on short-term endpoints and the CP
PoS	Posterior predictive probability of success
STE–PoS	Method for interim decision making based on short-term endpoints and the PoS
${\text{CP}}_{\text{cutoff}},{\text{PoS}}_{\text{cutoff}}$	Cutoffs for the PoS to terminate early in STE–CP and STE–PoS
PET	Probability of early termination
$n_{\text{I}},n_{\text{II}},n$	Sample size of the first stage, second stage, and total sample size
r	Threshold for number of long-term survivors for trial success
$s_{1}$	Threshold for number of short-term survivors to continue (in Kunz’s design)
K	Total number of measurement time points during the trial
k	Time point of measurement
$k^{\prime }$	Time point of last available measurement (relevant for prediction of long-term outcome)
$k^{*}$	Time point of the measurement of long-term endpoint, that is, the primary endpoint
$n_{k}$	Number of patients with the measurement at time point k
$x_{k}$	Number of survivors at time point k
$m_{k^{\prime }}$	Number of patients who were alive at $k^{\prime }$ but have pending assessments of subsequent time points
$y_{k^{\prime }}$	Number of patients among $m_{k^{\prime }}$ patients that is predicted to survive until $k^{*}$
$x_{k^{*}}$	Expected total number of long-term survivors at trial end
$p_{k|k-1}$	Probability to survive at least until k given survival at the previous time point
$p_{k^{*}|k^{\prime }}$	Probability of being long-term survivor given the last available measurement time point
$\hat{p}$	Maximum likelihood estimator of p
$P_{\text{prior}}(p)$	Prior distribution of p
$P_{\text{post}}(p)$	Posterior distribution of p

### 3.3. STE–CP for K measurement time points

The calculation of the CP requires the prediction of future data. At the time of the interim analysis, STE–CP assumes the estimated empirical trend for long-term survival probability for each individual, that is, the maximum likelihood estimate of the survival probabilities based on the data observed so far. The trial design aims to assess the outcome of each patient at $k^{*}$ time points. At the time of interim analysis, however, each patient $i\in \{1,\ldots ,n_{I}\}$ will only have available measurements up to time point $k_{i}^{\prime }\in \{1,\ldots ,k^{*}\}$ . Thus, each patient who is alive has an individual probability $p_{i,k^{*}|k_{i}^{\prime }}$ of being a survivor at the final measurement time point $k^{*}$ , which is calculated according to equation (2). For the analysis of this interim data, patients are grouped according to their number of available assessments, so that for each time point $k^{\prime }$ the number of patients who were alive at $k^{\prime }$ but have pending assessments at subsequent time points is denoted by $m_{k^{\prime }}$ . All $m_{k^{\prime }<k^{*}}$ patients are potential survivors at $k^{*}$ , while for $m_{k^{\prime }=k^{*}}$ patients survival is already known. Using Monte Carlo simulations, the number of these patients that will survive until long-term endpoint $k^{*}$ is sampled from a Binomial distribution, where the probability is the maximum likelihood estimate of the conditional survival probability derived in equation (2): $y_{k^{\prime }}∼\text{Binom}(m_{k^{\prime }},{\hat{p}}_{k^{*}|k^{\prime }})$ . To predict trial success, the expected total number of patients who will be alive at time point $k^{*}$ is calculated as $x_{k^{*}}=\sum_{k^{\prime }=0}^{k^{*}}y_{k^{\prime }}$ and compared to the critical value r . The percentage of Monte Carlo simulations in which $x_{k^{*}}>r$ , and thus the success of the trial is claimed, is the CP. The steps to conduct STE–CP are summarized in Algorithm 1.

Determination of the maximum likelihood estimate requires at least one patient with available data; if this is not the case, then an assumed value must be plugged in instead. Since power is calculated under the alternative hypothesis, we will use the a priori assumption for long-term survival under the alternative hypothesis, $p_{k^{*}\;,\;1}$ , and assume that for all time points the survival probabilities conditional on survival on the previous time point are equal, that is, $p_{1|0}=p_{2|1}=\cdots =p_{k^{*}|k^{*}-1}$ . This corresponds to constant hazards over time for a continuous survival endpoint. Given these assumptions, the missing conditional survival probabilities can be calculated as ${\hat{p}}_{k^{*}|k^{\prime }}=(p_{k^{*}\;,\;1}{)}^{1/(k^{*}-k^{\prime })}$ . Deriving the plug-in values from the null hypothesis instead would yield more conservative estimates of the CP. Depending on the sample size and the number of measurements, missing conditional probability estimates may occur relatively often and hence can have a considerable impact on the results. If there is any indication by previous data that another than this constant hazards assumption could be more suitable, then this should be considered. A sensitivity analysis should reveal the importance of the choice for the plugged-in estimate for a specific design.


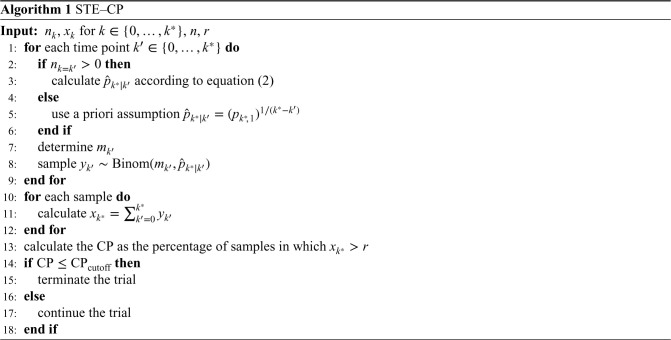


### 3.4. STE–PoS for K measurement time points

The calculation of the PoS requires the likelihood and a prior to deriving the posterior distribution of the conditional survival probabilities, from which then the posterior predictive distribution of long-term survivors can be generated. Given the likelihood in equation (1) and a prior distribution $P_{\text{prior}}(p_{k|k-1})$ for each $p_{k|k-1}$ , the posterior distribution of each $p_{k|k-1}$ is defined as:

(3) $$P_{\text{post}}(p_{k|k-1})\propto P_{\text{prior}}(p_{k|k-1})\times L\left(\left(p_{k|k-1}\right)|D\right)$$

The posterior distribution of $p_{k{}^{*}}$ given a patient was still alive at their last observation at time point $k^{\prime }$ arises as the product of the posterior distributions of the conditional survival probabilities for all time points $k^{\prime }+1,\ldots ,k{}^{*}$ :

(4) $$P_{\text{post}}\left(p_{k^{*}|k^{\prime }}\right)=\prod_{k=k^{\prime }+1}^{k^{*}}P_{\text{post}}(p_{k|k-1})$$

To obtain samples from the posterior distributions, we use Markov Chain Monte Carlo methods implemented in Stan^18^ via its interface to R^19^ and RStan.^20^ The remaining steps in STE-PoS are analogous to the ones in STE-CP, with a few differences. Provided the set of patients with pending outcomes $m_{k^{\prime }}$ as described in Section 3.3, the posterior predictive distribution of the number of long-term survivors at the end of the trial is sampled from a Binomial distribution, where the probability of survival is the posterior distribution of $p_{k^{*}|k^{\prime }}$ : $y_{k^{\prime }}∼\text{Binom}(m_{k^{\prime }},P_{\text{post}}(p_{k^{*}|k^{\prime }}))$ . To predict trial success, the expected number of long-term survivors is calculated as $x_{k^{*}}=\sum_{k^{\prime }=0}^{k^{*}}y_{k^{\prime }}$ and compared to the critical value r . The percentage of Monte Carlo simulations in which $x_{k^{*}}>r$ , and thus the success of the trial is claimed, is the PoS. The steps to conduct STE–PoS are also summarized in Algorithm 2.

In this small sample setting with strictly limited available information, the choice of the prior distributions is sensitive. In order to achieve a rather weakly informative prior distribution for the long-term survival probability, we choose negative log-Gamma distributions with shape parameter $1/k^{*}$ and rate parameter 1 for each conditional survival probability:

$$-\log\;(p_{k|k-1})∼\Gamma \left(1/k^{*},1\right)\text{ for }k\in \{1,\ldots ,k^{*}\}$$

This choice is motivated by the convenient property of the product of these distributions to arise as a Beta(α,1) distribution. Refer to Appendix 5 for more information. Therefore, since $p_{k^{*}}=\prod_{k=1}^{k^{*}}p_{k|k-1}$ , the prior distribution for the long-term survival probability is:

$$p_{k^{*}}∼\text{Beta}(\alpha ,1)$$

The parameter α is chosen such that the mean of the prior distribution of $p_{k^{*}}$ is equal to its assumed value under the alternative hypothesis. Alternatively, one could, for example, choose the assumed value under the null hypothesis to be more conservative.


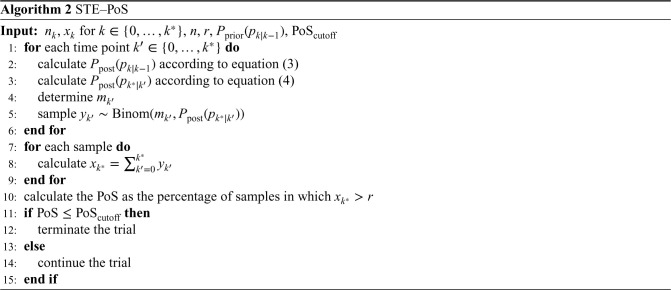


### 3.5. Cutoff value for stopping for futility

For decision making, both STE–CP and STE–PoS require to define a cutoff for the CP or the PoS, respectively, to stop the trial for futility. For both approaches, the PET is given by:

$$\begin{array}{rl} {\text{PET}}_{\text{STE-CP}} & =Pr(\text{CP}>{\text{CP}}_{\text{cutoff}})\text{ for STE-CP and}\\ {\text{PET}}_{\text{STE-PoS}} & =Pr(\text{PoS}>{\text{PoS}}_{\text{cutoff}})\text{ for STE-PoS} \end{array}$$

Saville et al.^10^ discuss possible justifications for choosing a specific cutoff value, and emphasize that the specific cutoff should typically be “unique to each trial” to be able to account for a variety of individual characteristics of the trial, such as ethical and risk/benefit considerations. In many clinical trials, it may be desirable to adhere to specific frequentist operating characteristics, such as type I error rate, power, or expected sample size.^11^ The cutoff can be defined such that specific requirements for these operating characteristics are met, which is ensured by means of simulations. In this paper, the cutoff is chosen such that the type I error rate of STE–CP and STE–PoS adheres to the predefined limit of 0.1. All other design parameters, that is, sample size of first and second stage, time point of interim analysis, and threshold r for rejecting the null hypothesis, are defined as in the optimal design by Kunz et al.^2^ The impact of the specific assumptions of the calibration on the trial design is in our experience rather small in most situations, and assessing operating characteristics under different cutoffs will be useful to investigate the impact of this choice. The most conservative cutoff may be chosen for strict type I error control, or an intermediate scenario may be preferred to balance type I error and power. In this study, we have taken the latter approach, and a detailed presentation of the impact of calibrations under other assumptions is provided in the Supplementary Material.

## 4. Simulation study

### 4.1. Simulation settings

To assess the operating characteristics of our new methods STE–CP and STE–PoS and to compare them to Kunz’s design, we conducted a simulation study. We assumed a phase II trial with long-term survival as a primary endpoint and the option to stop for futility. Long-term survival was defined as survival after 12 months and measurements were obtained every 3 months, so there were four measurement time points in total and $k^{*}=4$ . The simulations included three pairs of null and alternative hypotheses, in the following referred to as scenarios 1, 2, and 3. To calculate the optimal Kunz’s design, the first measurement time point after 3 months was defined as a short-term endpoint, $p_{\text{STE}}=p_{1}$ , and we assumed $p_{1}^{H_{0}}=0.7$ and $p_{1}^{H_{1}}=0.9$ for all scenarios. In scenario 1, the assumed rate of long-term survival was $p_{4}^{H_{0}}=0.127$ under the null hypothesis and $p_{4}^{H_{1}}=0.317$ under the alternative hypothesis, as in Kunz et al.^2^ With power 0.95 and type I error 0.10 , the optimal design has the design parameters $s_{1}=13$ , r=6 , $n_{I}=18$ , and n=38 . For scenario 2, the hypotheses were $p_{4}^{H_{0}}=0.25$ and $p_{4}^{H_{1}}=0.5$ , and the resulting design parameters were $s_{1}=12$ , r=10 , $n_{I}=17$ , and n=32 . For scenario 3, the hypotheses were $p_{4}^{H_{0}}=0.55$ and $p_{4}^{H_{1}}=0.8$ , and the resulting design parameters were $s_{1}=11$ , r=20 , $n_{I}=16$ , and n=31 . Kunz’s design was used as a reference design. Note that the design parameters of scenario 1 differed from the ones in Kunz et al.,^2^ because they had calibrated the design parameters under the assumption $p_{1}^{H_{0}}=1$ under the null hypothesis. We implemented STE–CP and STE–PoS, using the same sample sizes $n_{\text{I}}$ and n and the same critical threshold r . The only difference between Kunz’s design, STE–PoS, and STE–CP was the decision rule for early stopping. It would be possible to use a different timing of the interim analysis for STE–PoS and STE–CP, but it was chosen to be identical to Kunz’s design to make meaningful comparisons. Each simulation run produced one data set that was analyzed with all three methods, so all methods used the same data.

**Figure 1.**
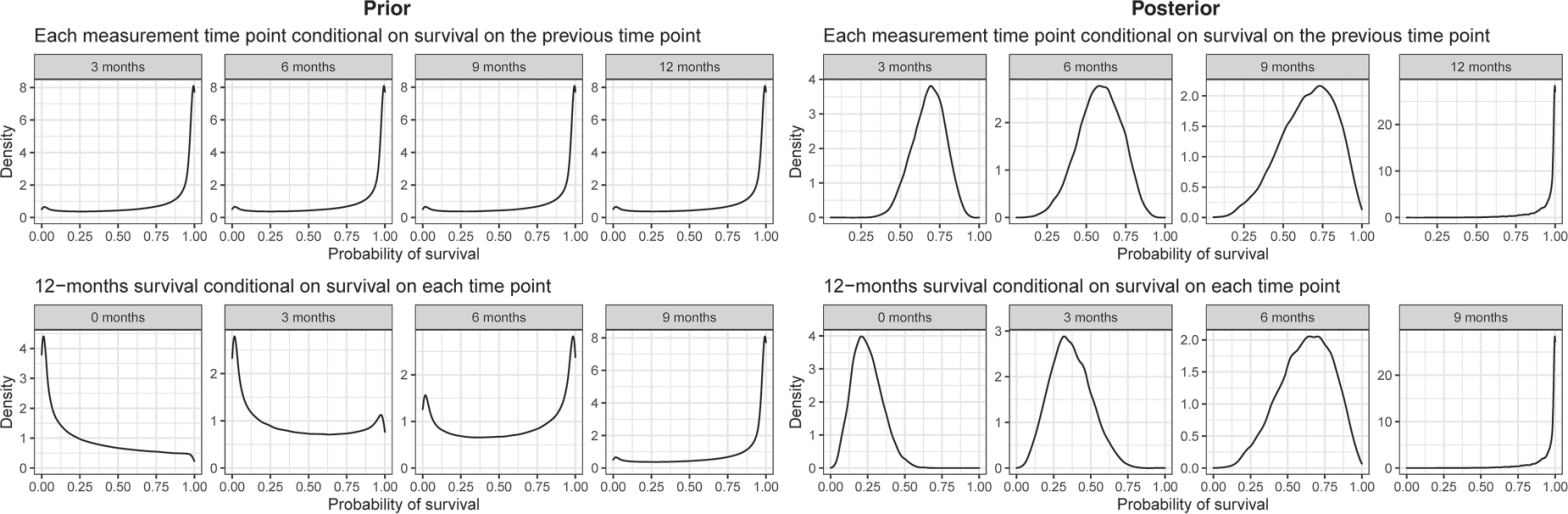
Visualization of the prior distributions used in the simulation and the posterior distribution for the example interim data presented in Table 2.

**Figure 2.**
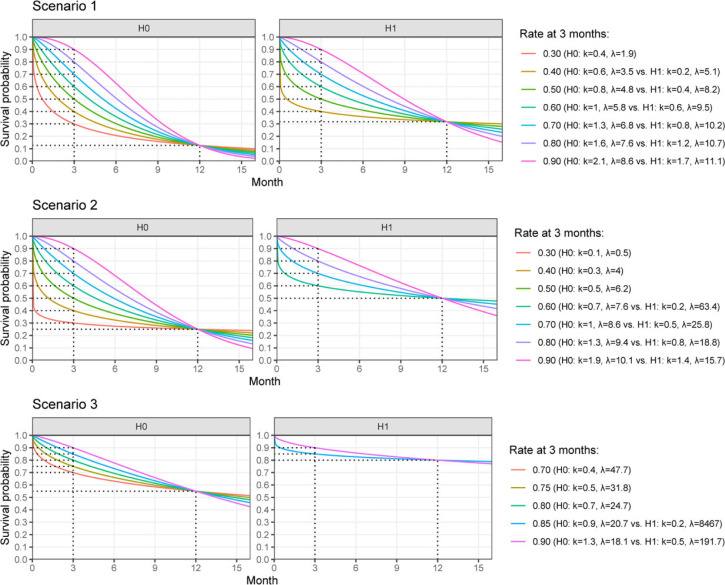
Null and alternative hypothesis for the long-term endpoint (after 12 months) with various scenarios for the short-term endpoint (after 3 months) and the corresponding Weibull survival functions with shape parameter k and scale parameter λ , which are used as data-generating mechanisms for the simulation study.

For all three designs, an interim analysis was conducted once $n_{\text{I}}$ patients had an observed 3-month outcome, and the trial stopped for futility or continued to stage 2 based on the respective decision rule. With STE–PoS, for scenario 1, the weakly informative prior $p_{4}∼\text{Beta}(0.464,1)$ was used, which has a mean of 0.317, which equals the 12-month survival rate under the alternative hypothesis. An illustration of this prior is provided in Figure 1. For scenarios 2 and 3, the priors $p_{4}∼\text{Beta}(1,1)$ and $p_{4}∼\text{Beta}(4,1)$ were used to achieve means 0.5 and 0.8, respectively. We considered various scenarios for the true $p_{1}$ between 0.9 and the lower limit (the short-term survival logically cannot be lower than the long-term survival). The time to event for each patient was simulated from a Weibull survival function with shape and rate parameters specified such that the corresponding scenario for $p_{4}$ and $p_{1}$ was met. All data-generating survival functions are visualized in Figure 2. Patient accrual per month followed a Poisson distribution with mean λ , for which there were four settings: 0.5, 1, 2, and 4. Each combination of simulation settings was simulated 100,000 times. The complete simulation code is made publicly available via GitHub: https://github.com/dariozchl/STE-CP-and-STE-PoS---code-for-Zocholl-et-al-2023-SMMR.

For illustration purposes, a single simulation run is summarized in Table 2. For this, time-to-event was sampled from a Weibull(κ=1.3,λ=6.8) distribution, corresponding to a 12-month survival rate of 0.317 , that is, alternative hypothesis being true, and a 3-month survival rate of 0.7 . Recruitment speed was 1 patient per month. In this example, the trial would have stopped for futility according to Kunz’s design, since the number of 3-month survivors was not higher than the critical threshold $s_{1}=13$ . However, both STE–CP and STE–PoS returned a relatively large probability to reject the null hypothesis at the end of the trial of 0.8064 and 0.7784, respectively, which was above the thresholds for continuation as will be seen in the next subsection. Therefore, in this example, both methods would have come to a different conclusion than Kunz’s design and would have recommended continuing to the second stage. For STE–PoS, the posterior distributions for this example data are visualized in Figure 1.

**Table 2.** Summary of example simulation run generated under scenario 1. Note that STE–PoS uses the whole posterior distribution, even though in this table only the posterior means are displayed.

				Being alive at measurement time in months (k)					
			Time under						
	Actual time	Time of	observation						
ID	to event	recruitment	at interim	0 ( k=0 )	3 ( k=1 )	6 ( k=2 )	9 ( k=3 )	12 ( $k=4=k^{*}$ )	$k^{\prime }$
1	4.61	0	28	Yes	Yes	No	–	–	–
2	0.66	0	28	Yes	No	–	–	–	–
3	3.70	1	27	Yes	Yes	No	–	–	–
4	13.51	6	22	Yes	Yes	Yes	Yes	Yes	4
5	6.86	7	21	Yes	Yes	Yes	No	–	–
6	12.94	7	21	Yes	Yes	Yes	Yes	Yes	4
7	18.71	8	20	Yes	Yes	Yes	Yes	Yes	4
8	8.64	11	17	Yes	Yes	Yes	No	–	–
9	0.90	11	17	Yes	No	–	–	–	–
10	2.91	11	17	Yes	No	–	–	–	–
11	5.11	20	8	Yes	Yes	No	–	–	–
12	0.81	20	8	Yes	No	–	–	–	–
13	4.85	21	7	Yes	Yes	No	–	–	–
14	2.20	22	6	Yes	No	–	–	–	–
15	15.21	22	6	Yes	Yes	Yes	Pending	Pending	2
16	4.77	22	6	Yes	Yes	No	–	–	–
17	0.81	24	4	Yes	No	–	–	–	–
18	13.04	25	3	Yes	Yes	Pending	Pending	Pending	1
$\frac{\text{\textbackslash{}\#survivors}}{\text{\textbackslash{}\#evaluable and alive at previous time point}}$			–	12/18	6/11	3/5	3/3		
				(Mean posterior) survival probabilities conditional on					
				being alive at the previous time point					
				${\hat{p}}_{k|k-1}$ and $E(P_{\text{post}}(p_{k^{*}|k-1}))$					
			Method	0 ( k=0 )	3 ( k=1 )	6 ( k=2 )	9 ( k=3 )	12 ( $k=4=k^{*}$ )	
			STE–CP	–	0.667	0.545	0.600	1	
			STE–PoS	–	0.679	0.584	0.659	0.946			
				(Mean) survival probabilities until month 12					
				conditional on being alive at this time point					
				${\hat{p}}_{k|k^{\prime }}$ and $E(P_{\text{post}}(p_{k^{*}|k^{\prime }}))$					
			Method	0 ( $k^{\prime }=0$ )	3 ( $k^{\prime }=1$ )	6 ( $k^{\prime }=2$ )	9 ( $k^{\prime }=3$ )	12 ( $k^{\prime }=4=k^{*}$ )	Pr(x>r)
			STE–CP	0.218	0.327	0.600	1	1	0.8064
			STE–PoS	0.247	0.364	0.623	0.946	1	0.7784

**Figure 3.**
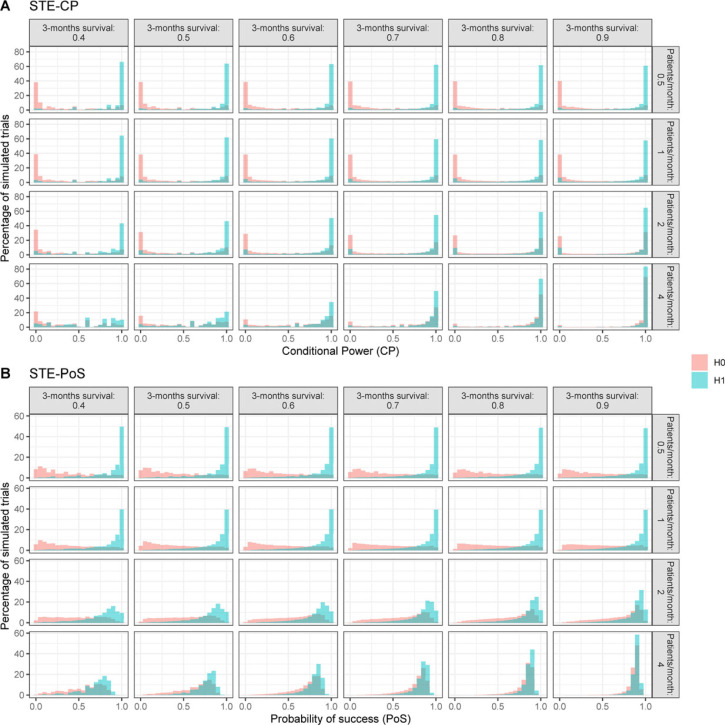
Distribution of CP according to STE–CP and the PoS according to STE–PoS for all pairs of null and alternative hypothesis within scenario 1. Results of scenarios 2 and 3 are presented in the Supplementary Material. CP: conditional power; PoS: probability of success; STE: short-term endpoints.

### 4.2. Simulation results

#### 4.2.1. Distribution of CP and PoS, and calibration of a cutoff

For the investigated simulation settings, the CP, as well as the PoS, tended toward small values in case the null hypothesis was true, and toward large values when the alternative hypothesis was true (scenario 1 shown in Figure 3, scenarios 2 and 3 shown in the Supplementary Material). This relation was stronger the slower the recruitment rate, since more long-term outcomes could be observed at the time of the interim analysis. Hence, the calculation could take into account more information. Although the general tendency was the same for STE–CP and STE–PoS, there were a few differences between the distributions. The distribution of the CP had a strong tendency toward the extreme values 0 and 1, while that of the PoS appeared smoother and produced less extreme values. This can be explained by the nature of the calculations within STE–CP and STE–PoS: for the calculation of the CP, a point estimate for the probability of an event was used to generate the new data, while for the calculation of the PoS the whole posterior distribution was used, which results for a specific interim analysis in a larger variance of the distribution of predicted outcomes. Therefore, the PoS will always be shrunken toward less extreme values compared to the CP. For very fast recruitment, that is, 4 patients per month on average, the distinction between null and alternative hypothesis became much less pronounced and the distributions were dominated by the prior assumptions rather than by the interim data. Combined with a high probability of 3 months survival, the PoS and CP were almost identical for the null and the alternative hypothesis scenario.

To define the cutoff for stopping for futility for each method, we assessed the distributions of PoS and CP for the scenario that the assumptions of Kunz’s design were correct and patient recruitment per month was Poisson distributed with mean λ=0.5 . Based on these assumptions, in scenario 1, the trial had a simulated type I error rate just below 0.10, if the cutoff value was 0.728 for CP and 0.734 for PoS. In scenario 2, the cutoff value was 0.560 for CP and 0.528 for PoS. In scenario 3, the cutoff value was 0.426 for CP and 0.468 for PoS. The results for calibrations under other assumptions are shown in the Supplementary Material.

#### 4.2.2. Probability of early termination

**Figure 4.**
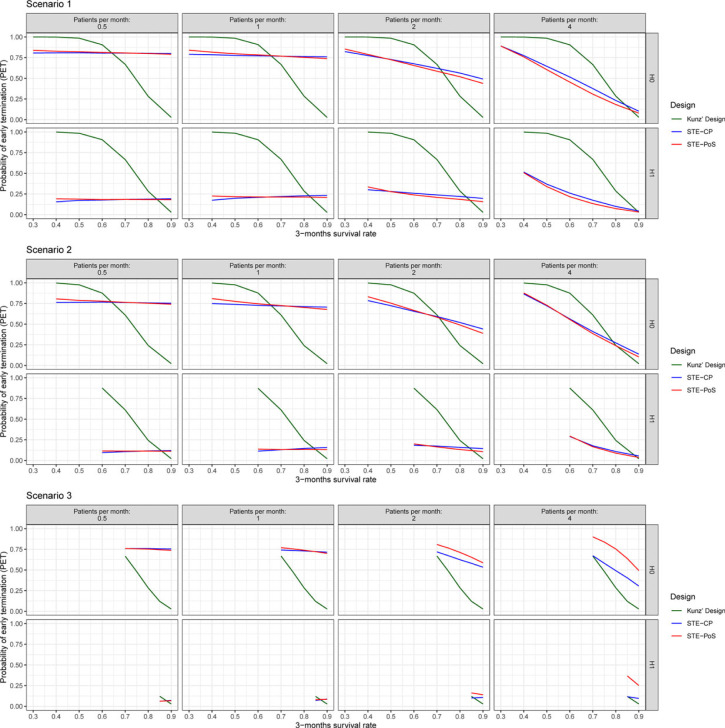
Probability of early termination after stage 1 of the investigated designs.

Using the established cutoff values, the PET for each scenario and each method was calculated (Figure 4, upper panel). In Kunz’s design, the PET was strongly dependent on the 3-month survival regardless of the true 12-month survival rate or patient recruitment speed. For both STE–CP and STE–PoS, the PET was very similar. It remained stable across all scenarios for the 3-month survival rates, if patient recruitment was rather slow, that is, λ of 0.5 or 1. Under the null hypothesis, the PET was large with values around 0.8, while it was rather small under the alternative hypothesis with values around 0.2. For faster recruitment, the PET became more dependent on the 3-month survival rate and more similar to the PET of Kunz’s design, and the difference between null and alternative hypothesis was less distinguished.

**Figure 5.**
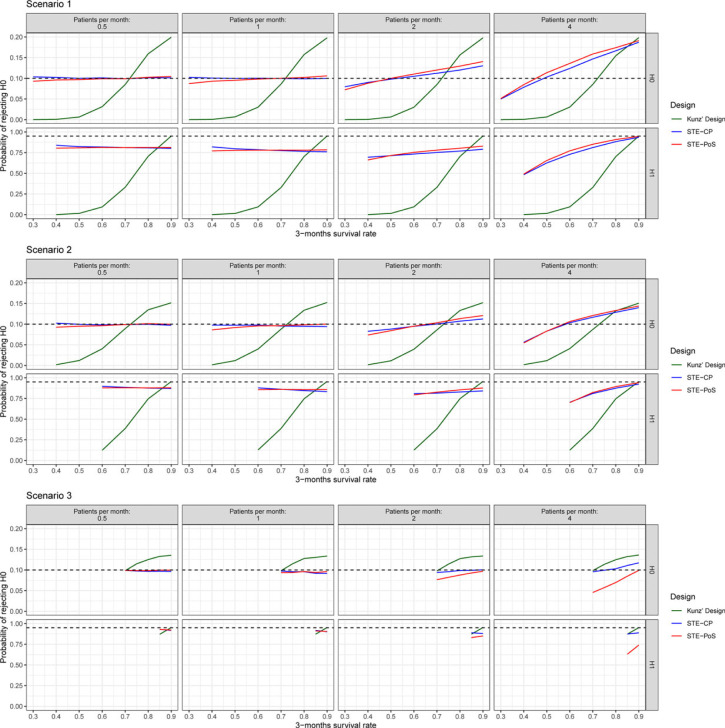
Type I error rate and power of the investigated designs. Black dashed lines indicate the prespecified type I error rate (0.10) and power (0.95) of Kunz’s design.

#### 4.2.3. Type I error rate and power

Type I error rate, defined as the rate of rejecting the null hypothesis when it was true, and power, defined as the rate of rejecting the null hypothesis when the alternative hypothesis was true, were calculated with the full data set, taking into account whether the trial has stopped early or continued to the second stage (Figure 5). Similar to the PET, the type I error rate and the power of Kunz’s design were dependent on the true 3-month survival rates but irrespective of the patient accrual. For STE–CP and STE–PoS they were stable across all scenarios for the 3-month survival rates if the patient accrual was slow. The faster the patient recruitment, the more power, and the type I error rate tended to become similar to Kunz’s design. Under slow recruitment of 0.5 or 1 patient per month, the type I error rate remained close to the desired 0.1 level for all scenarios with inflation of 0.006 in the most extreme case. Under fast patient recruitment of 4 patients per month, the type I error rate could not be controlled and was inflated up to 0.19 in the most extreme case for both STE–PoS and STE–CP, similar to Kunz’s design which was inflated to 0.20 in the same scenario. The power in scenario 1 with slow patient recruitment was approximately between 0.75 and 0.8, in scenario 2 between 0.85 and 0.90, and in scenario 3 between 0.90 and 0.93. If the assumptions for the 3-month survival rate were exactly correct, Kunz’s design of course met the requirement of 0.95 power. However, for smaller 3-month survival rates, the power advantage of STE–PoS and STE–CP was remarkable, particularly in scenarios 1 and 2. For example, in scenario 1 with a 3-month survival rate of 0.7 and accrual rate of 1 patient per month, Kunz’ design had a power of 0.33 compared to 0.78 with STE–PoS and 0.79 with STE–CP. If the 3-month survival rate was as low as 0.4, the power of Kunz’s design approximated 0 while the power of STE–PoS and STE–CP was still not affected. In scenario 3, the observed differences between the designs were much smaller, because the parameter space of the 3-month survival rate was very limited due to the high long-term survival rate under the null and alternative hypotheses.

#### 4.2.4. Informative priors

To explore the potential impact of informative priors in STE–PoS, we have additionally simulated scenario 1 with more informative prior distributions. In order to derive these priors, survival data of 10 patients was assumed to be available from a pilot study, of which 5 patients had survived at least up to 12 months, therefore supporting an optimistic expectation toward the trial’s outcome. The posterior distributions of the conditional survival probabilities were assessed. Using the R package *fitdistrplus*,^21^ Beta distributions were fitted to the posterior distributions, which were then specified as informative priors to re-analyze the simulations of scenario 1. To decide whether to stop for futility, the cutoff 0.734 was used for the PoS, which is the cutoff of the previous simulations with weakly informative priors. The results with informative priors were, in general, similar to those with weakly informative priors, with one important difference: the PET was consistently smaller, resulting in a consistently higher power (approximately 0.95 for slow recruitment) and higher type I error rate (approximately 0.15 for slow recruitment). Also worth mentioning is the slightly diminished stability across different short-term survival rates under all recruitment speeds, indicating that the informative priors do not seem to be robust against deviations from the assumed relations between short-term and long-term survival rates. Details about the data used for the prior and the full simulation results are provided in the Supplementary Material.

## 5. Discussion

Two-stage single-arm trials with the option to stop for futility are often planned according to Simon’s design. However, if the primary outcome takes a long time to be observed, such as 12-month survival, Simon’s design implies a long interruption of patient recruitment to conduct the interim analysis. Kunz et al.^2^ have offered an efficient solution by assessing a short-term endpoint, such as 3-month survival, at the interim to make the decision about stopping for futility. We showed that if the assumptions about the short-term endpoint are wrong, Kunz’s design could suffer from drastically increased PET under the alternative hypothesis and thus diminished power. We proposed two methods, STE–CP and STE–PoS, that provide decision rules for such trials based on short-term endpoints without requiring any additional strong assumptions. In this regard, they complement the existing design of Kunz et al.^2^ The methods apply the framework of CP and PoS, respectively. Since STE–PoS is embedded in the Bayesian framework, a definition of prior distributions is required. We have presented a default weakly informative prior distribution that can easily be adapted to be more optimistic or conservative.

The two proposed designs are both allowing for completely flexible timing of the interim analysis. In our simulations, the break between the two stages was of the same length as in Kunz’s design, and therefore considerably shorter than in Simon’s design, but that is not a requirement and it could also be shorter (or longer). The longer the break, the more patients will have complete follow-ups in the interim, so the more accurate the predictions will be. To correctly assess the operating characteristics of a specific trial, the length of the break must be prespecified in the planning stage. A (rather weak) assumption of the methods is that for each endpoint there should be at least one observation. If that is not the case, the survival probabilities need to be imputed, for example, by interpolating the assumed underlying survival curve. Our simulations have shown that assuming constant hazards over time work reliably also under non-constant hazards.

The simulations showed that STE–CP and STE–PoS closely control the type I error rate in all considered scenarios with slow patient recruitment and substantially improve power compared to Kunz’s design in many situations. The price is a loss of power in the narrow window where the true parameters of short-term and long-term survival are exactly or very close to as postulated in the design stage. If the assumptions about the short-term survival from the design stage were correct, STE–CP and STE–PoS had lower power than Kunz’s design (between 0.75 and 0.9 compared to 0.95 under Kunz’s design). The more one is off with the assumptions in the design phase about the short-term survival, the greater the advantage of the new methods over Kunz’s design, because the power of Kunz’s design will drop drastically (down to zero) while the power of STE–PoS and STE–CP is barely affected. We emphasize that this is only true for slow patient recruitment and that for trials with fast recruitment, the methods would not be applicable, because then none or only little information on the long-term endpoint would be available for the interim analysis. The extent to which a trial could potentially benefit from STE–CP and STE–PoS depends also on the hypothesis to be tested: if the long-term survival rate is assumed to be rather low, the parameter space of the short-term survival rate is rather large, and hence the benefit in terms of power and type I error rate could be quite large (as seen from scenario 1 in our simulations). If the long-term survival rate is greater, the parameter space of the short-term survival rates gets smaller, hence less power can be gained (as seen from scenario 2 and particularly scenario 3).

If prior information is available, one might want to express this by informative priors. We have presented a simple way of how this can be done with STE–PoS in principle. Adjusting the prior distributions to express certain prior information is easily done, and as expected optimistic informative priors led to decreased PET, and increased type I error rate and power. Interestingly, the stability across different short-term survival rates diminished compared to weakly informative priors, which is a sign of a lack of robustness against deviations from the assumed relations between short-term and long-term survival rates. We would like to emphasize that the origin of this work was the situation of extremely scarce information, so weakly informative priors will be more appropriate in the intended applications, and the formulation of informative priors will require additional research before applicable in practice.

The differences between the operating characteristics of STE–CP and STE–PoS (with weakly informative priors) were small, so they are more relevant on a philosophical level. The distribution of CP values under STE–CP showed a strong tendency toward extreme values close to or equal to 0 or 1 compared to the distribution of PoS values under STE–PoS, which can be explained by the fact that STE–CP uses a single-point estimate of the probability of survival to predict new data, while STE–PoS uses the whole posterior distribution.

One should note that we have considered binding stopping rules, so our results are only valid if one follows the stopping rule. There are good arguments to have a non-binding stopping rule, the most important one being able to react flexibly to information not considered in the stopping rule, such as adverse events.^22^ Nevertheless, the most popular design, Simon’s two-stage design, applies a binding stopping rule. Since it is impossible to quantify the operating characteristics under a non-binding stopping rule, calculations often assume no stopping under the null and a binding stopping rule under the alternative hypothesis, which is also how Kunz et al. have originally defined their design.^2^ If one would apply such non-binding stopping rules to the presented simulation study, then under the null hypothesis the decision making of our methods would reduce to Kunz’s design and type I error would be strictly controlled for all methods, while under the alternative hypothesis, all would remain as presented. Therefore, we want to emphasize that the improvement in type I error by our methods will vanish if non-binding rules are considered, and that the focus of this research was to improve the power under uncertainty about the expected short-term survival.

Another aspect is that for Kunz’s design, computationally efficient closed-form solutions exist, which implies an important practical advantage. For STE–PoS and STE–CP, calculations are much more time-consuming. Although it would be possible to calculate exact operating characteristics, the number of possible outcomes is too high to be computationally feasible even for the small sample size and the few measurement time points that were considered here. Therefore, we relied on simulations. For the same reason, searching for suitable designs in the whole parameter space does not seem to be feasible at the moment. Therefore, we suggest first finding the optimal Kunz design with a somewhat higher power than desired and then use this design as starting point for simulating the operating characteristics of STE–PoS or STE–CP.

When planning a trial, one should carefully consider whether the advantage of a faster conduct of the trial is worth the more complicated trial design compared to the relatively simple Simon’s two-stage design, which also has a completely stable type I error rate and power regardless of any other endpoints. In our discussions with clinical trial teams, the time advantage appeared often to be desirable, but the trade-off may be different for each situation. With this study, we hope to provide the required tools when facing the dilemma between an undesirably long interruption under Simon’s design and the risk of an underpowered study under Kunz’s design due to uncertainty about the short-term endpoint.

In principle, the presented methods are not limited to two-stage clinical trials with stopping for futility but could be applied to any setting in which one or more interim analyses are conducted and a survival endpoint with discrete measurement times is estimated. As they do not include a decision rule for the final analysis, such final decision rules need to be developed for the specific situation. Also, we have not considered the use of baseline covariates, as proposed recently in the context of randomized clinical trials.^8^ Baseline covariates could possibly be utilized to account for informative censoring, although the small sample sizes might make it challenging to achieve a proper adjustment for multiple variables, since in the first stage of such early phase clinical trials often only around 15 to 25 patients are recruited. A further option to improve STE–PoS could be to incorporate informative priors, for which we have suggested a possible workflow, but more thorough investigation, particularly of robust priors, will be required to support their application in practice.

## Supplemental Material

sj-pdf-1-smm-10.1177_09622802231188515 - Supplemental material for Using short-term endpoints to improve interim decision making and trial duration in two-stage phase II trials with nested binary endpoints Click here for additional data file. Supplemental material, sj-pdf-1-smm-10.1177_09622802231188515 for Using short-term endpoints to improve interim decision making and trial duration in two-stage phase II trials with nested binary endpoints by Dario Zocholl, Cornelia U. Kunz and Geraldine Rauch in Statistical Methods in Medical Research

## Appendices

### A Choice of prior

In Section 3.4, we described that a Beta(α,1) prior is used for the long-term survival probability $p_{k^{*}}$ because this distribution has the convenient property to arise as the product of negative log-Gamma distributions. To demonstrate the relationship between the Gamma distribution and the Beta(α,1) , assume Z∼Beta(α,1) , where Z is the product of K i.i.d. random variables, that is, $Z=\prod_{k=1}^{K}X_{k}$ . The probability density function of Z is $f(z)=\frac{z^{\alpha -1}}{B(\alpha ,1)}=\alpha z^{\alpha -1}$ . We then apply a negative natural logarithmic transformation to Z and formulate its moment-generating function:

$$\begin{array}{rl} M_{-\log\;(Z)}(t) & =E(e^{-t\log\;Z})\\ & =E(Z^{-t})\\ & =\int_{0}^{1}\alpha z^{\alpha -1-t}dz\\ & =\alpha \frac{1}{\alpha -t}z^{\alpha -t}{|}_{0}^{1}\\ & =\alpha \frac{1}{\alpha -t}1^{\alpha -t}\\ & =\frac{1}{1-t/\alpha }\\ & ={\left(1-t\alpha^{-1}\right)}^{-1} \end{array}$$

which can be recognized as the moment-generating function of a Gamma distributed random variable with shape parameter 1 and rate parameter α , that is, −log(Z)∼Γ(1,α) . Since the sum of K i.i.d. random Gamma variables follows a Gamma distribution with the same scale parameter and shape parameter 1/K , it follows that if $-\log\;(X_{k})∼\Gamma (1/K,\alpha )$ , then $\prod_{k=1}^{K}X_{k}=Z∼\text{Beta}(\alpha ,1)$ .
