# Supplementary material for: Using short-term endpoints to improve interim decision making and trial duration in two-stage phase II trials with nested binary endpoints
Source: Stat Methods Med Res. 2023 Jul 25;32(9):1749–65. doi: 10.1177/09622802231188515 (PMC10540486; doi:10.1177/09622802231188515)
Supplement: sj-pdf-1-smm-10.1177_09622802231188515 - Supplemental material for Using short-term endpoints to improve interim decision making and trial duration in two-stage phase II trials with nested binary endpoints [file sj-pdf-1-smm-10.1177_09622802231188515.pdf]

Supplementary materials for

Using short-term endpoints to improve interim decision making and trial duration in two-stage phase II trials with nested binary endpoints

1 Additional simulation results

This supplementary material file contains additional simulation results. First the distribution of the conditional power (CP) and posterior predictive probability of success (PoS) is shown for 100,000 simulation of all simulation settings within scenario 2 and 3. For description of the simulation parameters, refer to the main document. Secondly, we present the results of the exploratory application of an informative prior with STE-PoS.

1.1 Distribution of CP and PoS

For both scenarios 2 and 3, it was observed that regardless of the true rate of the short-term survival, the values of both CP and PoS tended towards smaller values under the null hypothesis and towards larger values under the alternative hypothesis (Figure S1). This relationship was stronger, when patient recruitment was slow. For fast patient recruitment of 4 patients per month, null and alternative hypothesis could barely be distinguished anymore. These observations were very similar to what was observed in scenario 1 in the main document.

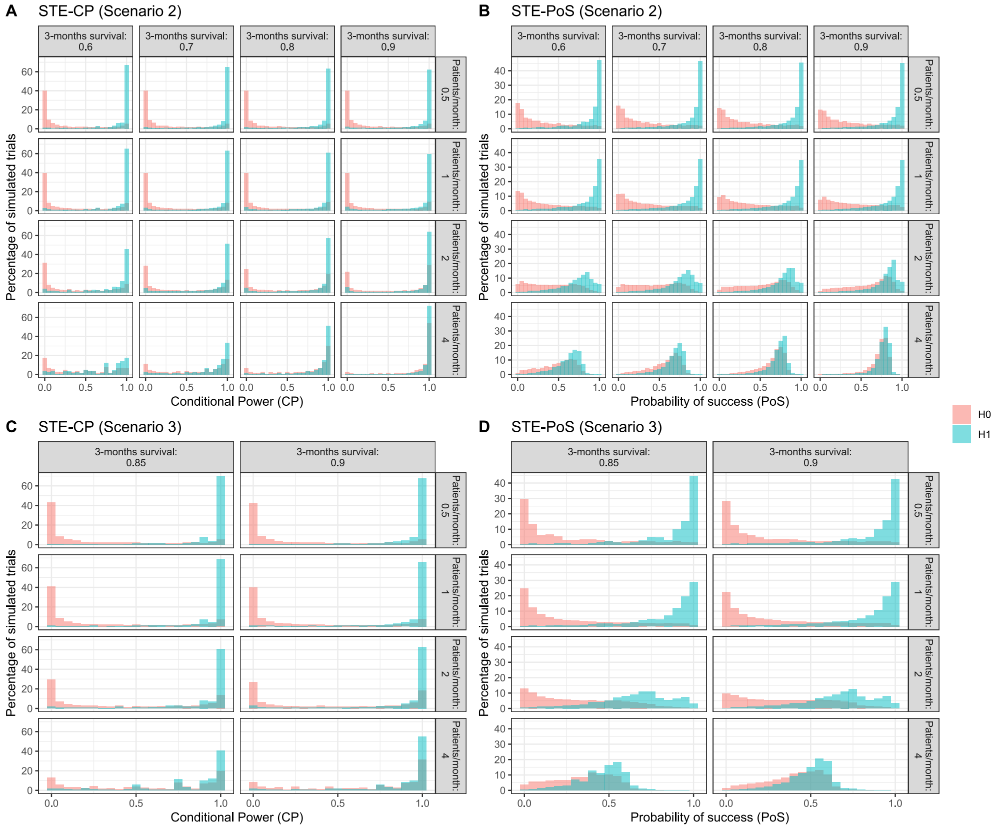

Figure S1: Distribution of CP according to STE-CP and the PoS according to STE-PoS under the null and alternative hypothesis for all simulation settings within scenario 2 and 3.

1.2 Sensitivity analysis for different cutoffs

By assuming different data-generating mechanisms, one would obtain different cutoff values to decide whether to stop for futility. To assess the sensitivity of the simulation results with regard to the choice of the cutoff value calibration, we have calibrated the respective cutoffs under all considered data-generating mechanisms with slow patient recruitment (0.5 patients per month). Note that with fast patient recruitment, the methods could not be meaningfully applied anyways, so calibration of the cutoff under such an assumption would not be useful. The operating characteristics under all cutoff calibrations are shown in tables tables S2 to S11. For scenario 1 the spread of possible cut-offs was the largest, because the most divergent correlations were possible, but the impact was still rather small: the cut-offs for PoS ranged from 0.721 to 0.758, for CP they ranged from 0.745 to 0.761. For STE-PoS, the two most extreme cut-offs could result in a difference of up to 0.0086 in type I error rate, and up to 0.024 in power. For most calibrations, the difference between the two extreme cut-offs was much smaller, though. For STE-CP, the impact was up to 0.0025 in type I error rate and up to 0.0067 in power. In our view, this potential impact can be considered as quite small, particularly considering the comparatively huge impact of various correlations between endpoints in the reference design by Kunz' et al. However, it is not guaranteed to be necessarily negligible. In practice, assessing operating characteristics under different cut-offs is recommended, and the most conservative cut-off may be chosen for strict type I error control, or an intermediate scenario may be preferred to balance type I error and power.

1.3 Exploratory application of informative priors

In the main document, we described a simulation study, in which the information about survival rates of 10 patients was transformed into informative prior distributions and the results of the simulations were then re-calculated. Here we show in more detail the data that was used to inform the prior distributions, how it was done, and what the results with this prior. Still, we would like to emphasize that this analysis is purely exploratory in the sense that these informative prior distributions are naive and not robustified in any way, i.e., in the event of a prior-data conflict there is no possibility within the model to identify or even react to it. Our analysis rather demonstrates how the priors can be adjusted to be more informative in a general sense and how this impacts the operating characteristics of the method. The focus of our work was to establish methods for the situation of extremely scarce information, and to formulate more robust priors, additional research will be needed.

The data used to inform the prior distributions is displayed in Table S1. The data contained 10 patients whose survival outcomes had been observed for 12 months. Of these 10 patients, 5 had survived after 12 months. Keeping in mind that under the alternative hypothesis the probability of 12-months survival was 0.317, this prior data supports an optimistic expectation towards the trial's outcome. Th data was analyzed with the Bayesian model and the weakly informative prior distributions for scenario 1 as described in the main document. To the resulting posterior distributions, Gamma distributions were fitted with the R package *fitdistrplus*, which were then used as new prior distributions for the actual trial. The parameters can be seen in Table S1. Additionally, we also fitted a Beta distribution to the posterior distribution of $p_{4|0}$, which ideally should coincide with the product of the Gamma distributions. Assessing the distributions graphically in Figure S2, it can be seen that the distributions fitted quite closely. As a consequence, the following informative priors were used to repeat the simulations of scenario 1: $-\log(p_{1|0}) \sim \Gamma(0.25, 11.0)$, $-\log(p_{2|1}) \sim \Gamma(3.22, 9.26)$, $-\log(p_{3|2}) \sim \Gamma(1.24, 7.43)$, $-\log(p_{4|3}) \sim \Gamma(1.24, 6.42)$.

Just as in the simulations in the main document, a 100,000 simulations were evaluated for each setting. The distribution of the PoS with informative priors for all simulation settings within scenario 1 can be seen in Figure S3. Unsurprisingly, compared to the distribution with weakly informative priors in the main document, the PoS values here had a much stronger tendency towards 1 in general. The other observations remain the same: PoS values tended towards smaller values under the null hypothesis and vice versa under the alternative hypothesis. For slow patient recruitment it worked well, but for fast patient recruitment of 4 patients per month, the distributions of PoS under null and alternative hypothesis became very similar.

The operating characteristics were also impacted by the informative prior as expected (Figure S4). The PET was consistently lower with informative priors, and power and type I error rate were consistently higher. For slow patient recruitment, the operating characteristics were again stable across various short-term survival rates. However, this stability seemed to be reduced compared to the approach with weakly informative priors or with conditional power. This can be well recognized for the type I error rate with slow patient recruitment, where the type I error rate increased for larger short-term survival rates from 0.150 to 0.175, indicating a lack of robustness against deviations from the assumed relations between short-term and long-term survival rates. In other words, the informative prior distributions seem to have induced an expectation about a specific shape of the survival function, and if the data deviated from it, the results were affected. It should noted that power could be greatly increased to approximately 0.95 in almost all scenarios but the type I error rate could not be maintained at 0.10 and increased to at least 0.15 and up to 0.20.

*ID	actual *time to event	being alive at measurement time in months (k)				
		0 ($k = 0$)	3 ($k = 1$)	6 ($k = 2$)	9 ($k = 3$)	12 ($k = 4 = k^*$)
1	6.84	yes	yes	yes	no	-
2	-	yes	yes	yes	yes	yes
3	10.98	yes	yes	yes	yes	no
4	-	yes	yes	yes	yes	yes
5	3.71	yes	yes	no	-	-
6	-	yes	yes	yes	yes	yes
7	-	yes	yes	yes	yes	yes
8	4.28	yes	yes	no	-	-
9	3.50	yes	yes	no	-	-
10	-	yes	yes	yes	yes	yes
	survivors	-	10/10	7/10	6/7	5/6

	mean posterior survival probabilities conditional on being alive at the previous time point $E\left(P_{\text{post}}\left(p_{k^*\|k-1}\right)\right)$				
	0 ($k = 0$)	3 ($k = 1$)	6 ($k = 2$)	9 ($k = 3$)	12 ($k = 4 = k^*$)
STE-PoS	-	0.978	0.719	0.855	0.836

	Parameters of negative logarithmic Gamma distributions when fitted to 200,000 posterior samples					
	0<br>-	3<br>$P_{\text{post}}(p_{1\|0})$	3<br>$P_{\text{post}}(p_{2\|1})$	9<br>$P_{\text{post}}(p_{3\|2})$	12<br>$P_{\text{post}}(p_{4\|3})$	(Beta distr.)<br>$P_{\text{post}}(p_{4\|0})$
shape	-	0.25	3.22	1.24	1.24	$\alpha = 6.09$
rate	-	11.0	9.26	7.43	6.42	$\beta = 6.03$

Table S1: Data used to generate the informative priors.

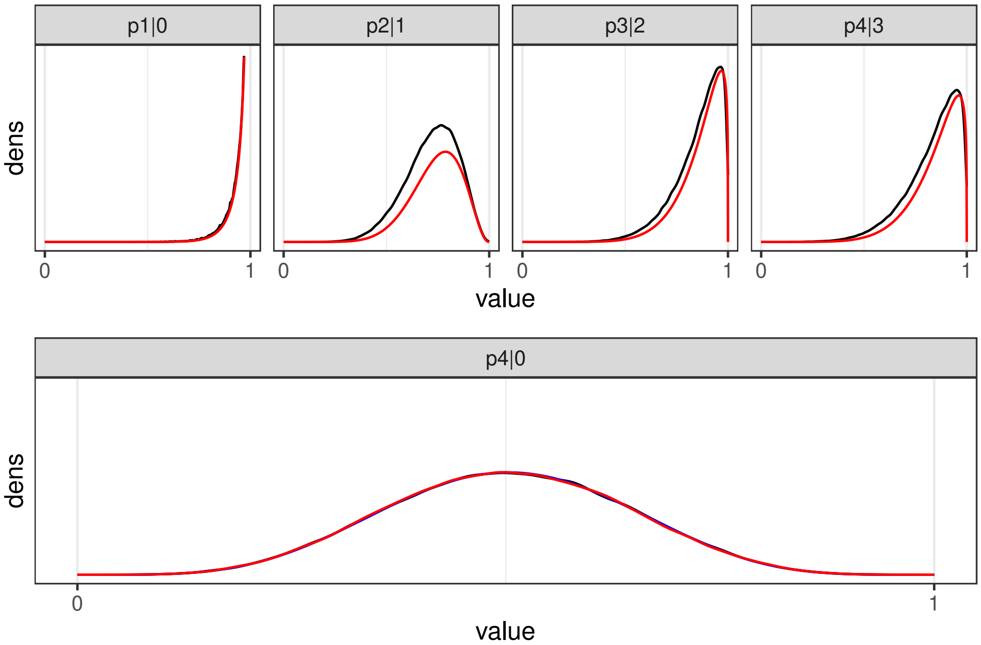

Figure S2: Densities (black lines) of 200,000 samples from the posterior distributions based on the data from Table S1. Red lines represent the fitted negative logarithmic Gamma distributions, for $p_{4|0}$ red line represents the product of random samples of these Gamma distributions. The blue line for $p_{4|0}$ is the result of fitting a Beta distribution to $P_{\text{post}}(p_{4|0})$ directly; if the three lines are hard to distinguish, it is because of the large overlap.

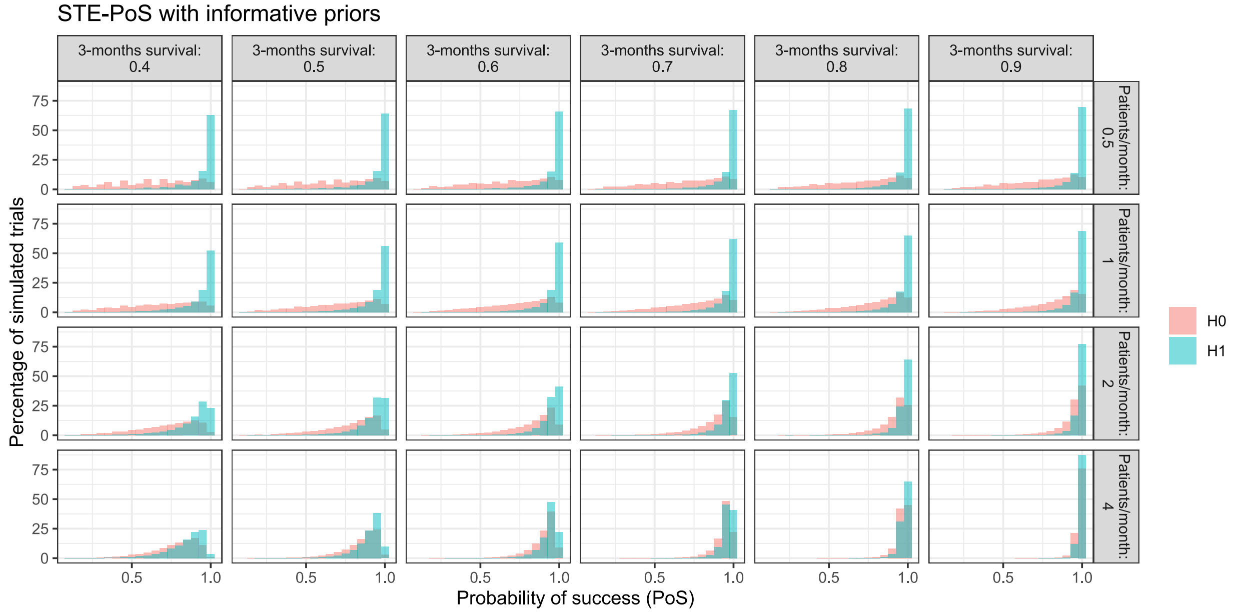

Figure S3: Distribution of the PoS according to STE-PoS with informative priors under the null and alternative hypothesis for all simulation settings within scenario 1.

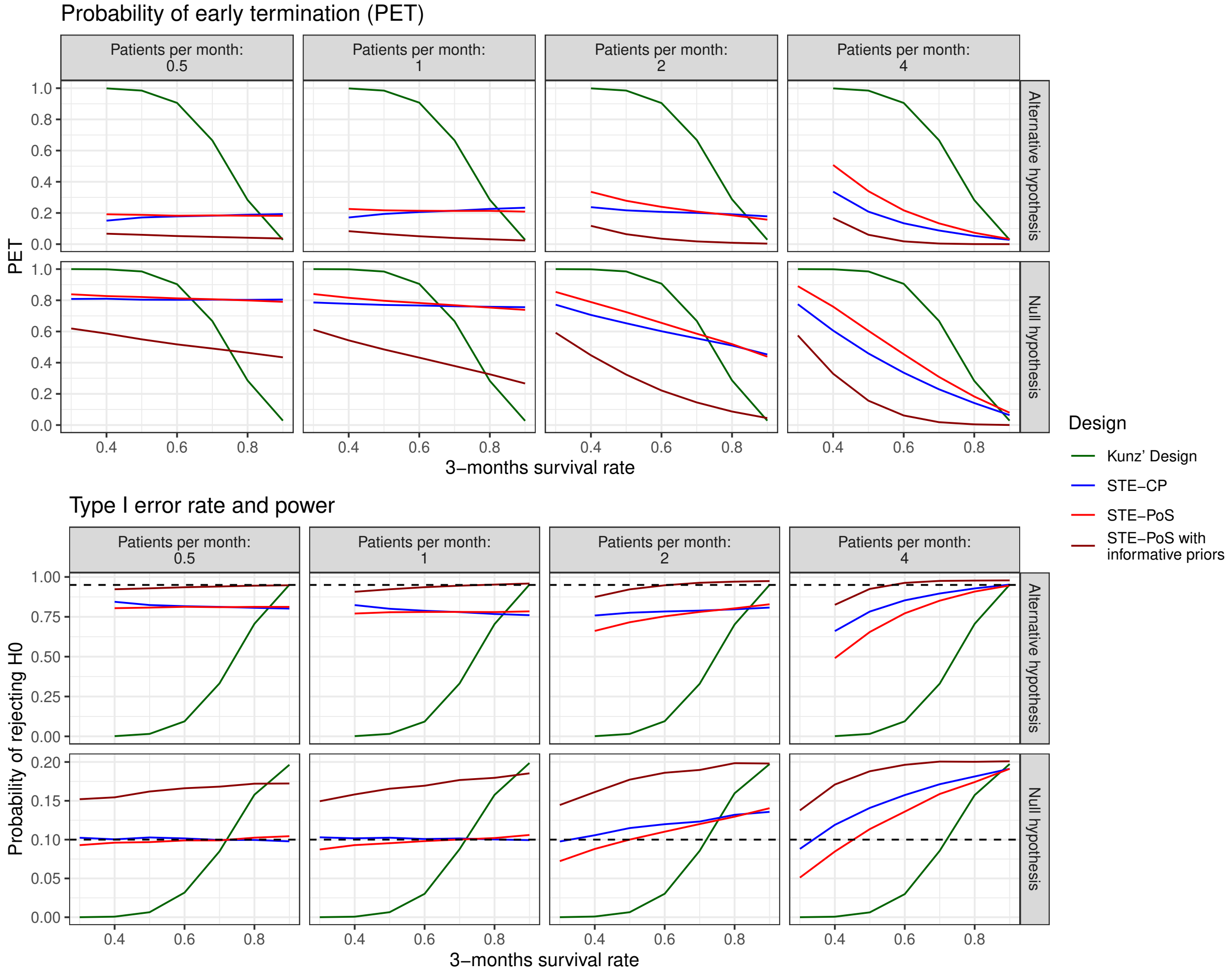

Figure S4: Operating characteristics as presented in the main document, supplemented by STE-PoS with informative priors.

simulation setting ID	patients per month	true p_1	hypothesis	design	Probability of early termination: Cutoff calibrated under short-term rate p_1 0.4	0.5	0.6	0.7	0.8	0.9
1	0.5	0.3	H0	STE-CP	0.804	0.805	0.807	0.807	0.808	0.810
				STE-PoS	0.831	0.833	0.838	0.838	0.846	0.851
2		0.4		STE-CP	0.806	0.807	0.809	0.809	0.812	0.815
				STE-PoS	0.819	0.821	0.827	0.827	0.835	0.840
3		0.5		STE-CP	0.807	0.808	0.811	0.811	0.814	0.818
				STE-PoS	0.813	0.815	0.821	0.821	0.830	0.835
4		0.6		STE-CP	0.802	0.803	0.807	0.807	0.812	0.816
				STE-PoS	0.803	0.806	0.812	0.812	0.822	0.828
5		0.7		STE-CP	0.802	0.804	0.808	0.808	0.813	0.816
				STE-PoS	0.798	0.800	0.807	0.807	0.817	0.823
6		0.8		STE-CP	0.800	0.801	0.805	0.805	0.810	0.814
				STE-PoS	0.789	0.792	0.799	0.799	0.809	0.816
7		0.9		STE-CP	0.797	0.798	0.803	0.803	0.809	0.813
				STE-PoS	0.781	0.784	0.790	0.790	0.800	0.807
8	0.5	0.4	H1	STE-CP	0.154	0.155	0.156	0.156	0.157	0.159
				STE-PoS	0.183	0.185	0.192	0.192	0.201	0.207
9		0.5		STE-CP	0.171	0.172	0.174	0.174	0.176	0.178
				STE-PoS	0.181	0.183	0.188	0.188	0.196	0.201
10		0.6		STE-CP	0.175	0.176	0.178	0.178	0.182	0.185
				STE-PoS	0.175	0.177	0.182	0.182	0.191	0.197
11		0.7		STE-CP	0.182	0.184	0.187	0.187	0.191	0.194
				STE-PoS	0.176	0.179	0.184	0.184	0.194	0.199
12		0.8		STE-CP	0.185	0.187	0.190	0.190	0.195	0.198
				STE-PoS	0.174	0.176	0.182	0.182	0.192	0.197
13		0.9		STE-CP	0.190	0.191	0.195	0.195	0.201	0.205
				STE-PoS	0.174	0.176	0.182	0.182	0.191	0.197
14	1	0.3	H0	STE-CP	0.789	0.791	0.792	0.792	0.794	0.797
				STE-PoS	0.831	0.834	0.840	0.840	0.848	0.854
15		0.4		STE-CP	0.783	0.784	0.786	0.786	0.790	0.793
				STE-PoS	0.807	0.810	0.816	0.816	0.825	0.832
16		0.5		STE-CP	0.774	0.776	0.778	0.778	0.783	0.787
				STE-PoS	0.786	0.789	0.797	0.797	0.808	0.814
17		0.6		STE-CP	0.769	0.771	0.775	0.775	0.780	0.784
				STE-PoS	0.773	0.776	0.783	0.783	0.794	0.801
18		0.7		STE-CP	0.765	0.766	0.770	0.770	0.776	0.779
				STE-PoS	0.758	0.761	0.768	0.768	0.780	0.787
19		0.8		STE-CP	0.760	0.762	0.765	0.765	0.771	0.774
				STE-PoS	0.742	0.745	0.753	0.753	0.766	0.773
20		0.9		STE-CP	0.758	0.759	0.763	0.763	0.768	0.772
				STE-PoS	0.727	0.730	0.739	0.739	0.751	0.759
21	0.5	0.4	H1	STE-CP	0.174	0.174	0.175	0.175	0.178	0.182
				STE-PoS	0.215	0.218	0.226	0.226	0.237	0.244
22		0.5		STE-CP	0.196	0.197	0.199	0.199	0.203	0.206
				STE-PoS	0.208	0.211	0.217	0.217	0.227	0.234
23		0.6		STE-CP	0.207	0.208	0.211	0.211	0.216	0.219
				STE-PoS	0.205	0.207	0.214	0.214	0.225	0.232
24		0.7		STE-CP	0.217	0.218	0.222	0.222	0.227	0.230
				STE-PoS	0.204	0.206	0.213	0.213	0.224	0.231
25		0.8		STE-CP	0.226	0.227	0.231	0.231	0.236	0.239
				STE-PoS	0.204	0.206	0.213	0.213	0.224	0.231
26		0.9		STE-CP	0.231	0.232	0.235	0.235	0.240	0.243
				STE-PoS	0.200	0.202	0.209	0.209	0.219	0.226

Table S2: Probability of early termination (PET) for scenario 1 with 0.5 and 1 patient(s) per month under various cutoff-calibrations.

simulation setting ID	patients per month	true p_1	hypothesis	design	Probability of early termination: Cutoff calibrated under short-term rate p_1 0.4	0.5	0.6	0.7	0.8	0.9
27	2	0.3	H0	STE-CP	0.822	0.823	0.824	0.824	0.833	0.843
				STE-PoS	0.841	0.845	0.854	0.854	0.866	0.872
28		0.4		STE-CP	0.773	0.774	0.777	0.777	0.785	0.791
				STE-PoS	0.772	0.777	0.789	0.789	0.804	0.813
29		0.5		STE-CP	0.725	0.726	0.732	0.732	0.741	0.745
				STE-PoS	0.706	0.711	0.724	0.724	0.743	0.754
30		0.6		STE-CP	0.671	0.674	0.680	0.680	0.689	0.692
				STE-PoS	0.636	0.641	0.655	0.655	0.675	0.688
31		0.7		STE-CP	0.616	0.619	0.624	0.624	0.631	0.635
				STE-PoS	0.567	0.573	0.586	0.586	0.605	0.618
32		0.8		STE-CP	0.560	0.562	0.567	0.567	0.573	0.576
				STE-PoS	0.502	0.506	0.519	0.519	0.538	0.550
33		0.9		STE-CP	0.489	0.490	0.494	0.494	0.500	0.503
				STE-PoS	0.423	0.427	0.439	0.439	0.456	0.468
34	2	0.4	H1	STE-CP	0.300	0.301	0.304	0.304	0.319	0.335
				STE-PoS	0.314	0.320	0.335	0.335	0.355	0.366
35		0.5		STE-CP	0.278	0.279	0.284	0.284	0.293	0.298
				STE-PoS	0.261	0.266	0.278	0.278	0.295	0.306
36		0.6		STE-CP	0.255	0.257	0.263	0.263	0.270	0.274
				STE-PoS	0.225	0.229	0.239	0.239	0.255	0.266
37		0.7		STE-CP	0.235	0.237	0.241	0.241	0.247	0.250
				STE-PoS	0.197	0.200	0.209	0.209	0.223	0.233
38		0.8		STE-CP	0.218	0.219	0.223	0.223	0.227	0.229
				STE-PoS	0.174	0.177	0.185	0.185	0.197	0.205
39		0.9		STE-CP	0.194	0.196	0.198	0.198	0.202	0.204
				STE-PoS	0.148	0.150	0.157	0.157	0.168	0.175
40	4	0.3	H0	STE-CP	0.890	0.891	0.893	0.893	0.898	0.900
				STE-PoS	0.870	0.876	0.891	0.891	0.905	0.914
41		0.4		STE-CP	0.770	0.772	0.781	0.781	0.794	0.798
				STE-PoS	0.727	0.737	0.759	0.759	0.784	0.802
42		0.5		STE-CP	0.636	0.641	0.655	0.655	0.674	0.679
				STE-PoS	0.568	0.579	0.604	0.604	0.634	0.661
43		0.6		STE-CP	0.507	0.512	0.525	0.525	0.541	0.546
				STE-PoS	0.422	0.431	0.454	0.454	0.484	0.510
44		0.7		STE-CP	0.371	0.374	0.380	0.380	0.388	0.393
				STE-PoS	0.283	0.290	0.309	0.309	0.334	0.356
45		0.8		STE-CP	0.231	0.232	0.234	0.234	0.239	0.243
				STE-PoS	0.167	0.171	0.183	0.183	0.202	0.216
46		0.9		STE-CP	0.102	0.102	0.105	0.105	0.110	0.113
				STE-PoS	0.071	0.073	0.079	0.079	0.088	0.095
47	4	0.4	H1	STE-CP	0.511	0.513	0.520	0.520	0.534	0.538
				STE-PoS	0.467	0.480	0.507	0.507	0.537	0.561
48		0.5		STE-CP	0.361	0.365	0.379	0.379	0.399	0.404
				STE-PoS	0.306	0.316	0.340	0.340	0.369	0.395
49		0.6		STE-CP	0.252	0.256	0.269	0.269	0.285	0.289
				STE-PoS	0.194	0.201	0.217	0.217	0.241	0.262
50		0.7		STE-CP	0.171	0.173	0.178	0.178	0.185	0.188
				STE-PoS	0.118	0.123	0.133	0.133	0.150	0.165
51		0.8		STE-CP	0.099	0.099	0.101	0.101	0.103	0.105
				STE-PoS	0.065	0.067	0.073	0.073	0.082	0.090
52		0.9		STE-CP	0.044	0.044	0.045	0.045	0.047	0.048
				STE-PoS	0.028	0.029	0.032	0.032	0.037	0.040

Table S3: Probability of early termination (PET) for scenario 1 with 2 and 4 patients per month under various cutoff-calibrations.

simulation setting ID	patients per month	true p_1	hypothesis	design	Probability of early termination: Cutoff calibrated under short-term rate p_1 0.4	0.5	0.6	0.7	0.8	0.9
53	0.5	0.4	H0	STE-CP	0.762	0.763	0.765	0.776	0.779	0.777
				STE-PoS	0.779	0.781	0.786	0.805	0.816	0.809
54		0.5		STE-CP	0.763	0.764	0.767	0.778	0.781	0.779
				STE-PoS	0.767	0.770	0.774	0.787	0.795	0.790
55		0.6		STE-CP	0.767	0.768	0.770	0.779	0.782	0.780
				STE-PoS	0.760	0.762	0.766	0.777	0.785	0.780
56		0.7		STE-CP	0.761	0.762	0.764	0.772	0.776	0.773
				STE-PoS	0.747	0.749	0.752	0.763	0.771	0.766
57		0.8		STE-CP	0.757	0.758	0.760	0.768	0.774	0.771
				STE-PoS	0.736	0.738	0.741	0.752	0.762	0.756
58		0.9		STE-CP	0.754	0.755	0.757	0.768	0.775	0.770
				STE-PoS	0.723	0.725	0.728	0.741	0.751	0.744
59	0.5	0.6	H1	STE-CP	0.095	0.095	0.096	0.102	0.104	0.103
				STE-PoS	0.101	0.102	0.104	0.115	0.121	0.118
60		0.7		STE-CP	0.107	0.108	0.109	0.115	0.117	0.116
				STE-PoS	0.103	0.104	0.106	0.113	0.118	0.115
61		0.8		STE-CP	0.116	0.116	0.117	0.122	0.125	0.123
				STE-PoS	0.104	0.105	0.107	0.113	0.118	0.115
62		0.9		STE-CP	0.120	0.121	0.122	0.128	0.132	0.130
				STE-PoS	0.102	0.103	0.105	0.112	0.118	0.114
63	1	0.4	H0	STE-CP	0.748	0.749	0.751	0.758	0.760	0.758
				STE-PoS	0.789	0.791	0.795	0.809	0.818	0.813
64		0.5		STE-CP	0.740	0.741	0.743	0.752	0.756	0.754
				STE-PoS	0.756	0.758	0.762	0.775	0.784	0.778
65		0.6		STE-CP	0.727	0.728	0.730	0.739	0.744	0.741
				STE-PoS	0.726	0.728	0.731	0.746	0.756	0.749
66		0.7		STE-CP	0.721	0.722	0.724	0.733	0.738	0.735
				STE-PoS	0.703	0.706	0.709	0.725	0.736	0.729
67		0.8		STE-CP	0.712	0.713	0.715	0.723	0.729	0.725
				STE-PoS	0.677	0.680	0.684	0.701	0.713	0.705
68		0.9		STE-CP	0.706	0.708	0.710	0.718	0.724	0.720
				STE-PoS	0.653	0.656	0.660	0.677	0.690	0.683
69	1	0.6	H1	STE-CP	0.113	0.114	0.115	0.119	0.121	0.120
				STE-PoS	0.124	0.125	0.128	0.138	0.145	0.140
70		0.7		STE-CP	0.130	0.130	0.132	0.137	0.140	0.138
				STE-PoS	0.120	0.121	0.123	0.132	0.139	0.134
71		0.8		STE-CP	0.146	0.146	0.147	0.153	0.157	0.154
				STE-PoS	0.120	0.121	0.123	0.133	0.141	0.136
72		0.9		STE-CP	0.157	0.158	0.159	0.165	0.169	0.166
				STE-PoS	0.118	0.120	0.122	0.131	0.139	0.134

Table S4: Probability of early termination (PET) for scenario 2 with 0.5 and 1 patient(s) per month under various cutoff-calibrations.

simulation setting ID	patients per month	true p_1	hypothesis	design	Probability of early termination					
					Cutoff calibrated under short-term rate p_1					
					0.4	0.5	0.6	0.7	0.8	0.9
73	2	0.4	H0	STE-CP	0.785	0.787	0.790	0.795	0.798	0.796
				STE-PoS	0.805	0.807	0.812	0.831	0.845	0.837
74		0.5		STE-CP	0.724	0.727	0.730	0.737	0.742	0.739
				STE-PoS	0.721	0.724	0.730	0.753	0.769	0.759
75		0.6		STE-CP	0.656	0.658	0.661	0.669	0.676	0.672
				STE-PoS	0.634	0.638	0.643	0.666	0.684	0.673
76		0.7		STE-CP	0.593	0.594	0.596	0.605	0.612	0.607
				STE-PoS	0.552	0.555	0.561	0.583	0.602	0.590
77		0.8		STE-CP	0.520	0.521	0.523	0.531	0.536	0.532
				STE-PoS	0.458	0.462	0.467	0.490	0.508	0.496
78		0.9		STE-CP	0.442	0.443	0.445	0.452	0.456	0.453
				STE-PoS	0.361	0.364	0.369	0.389	0.405	0.395
79	2	0.6	H1	STE-CP	0.185	0.187	0.191	0.195	0.198	0.196
				STE-PoS	0.174	0.176	0.181	0.201	0.217	0.207
80		0.7		STE-CP	0.175	0.176	0.178	0.183	0.187	0.184
				STE-PoS	0.143	0.145	0.149	0.164	0.176	0.169
81		0.8		STE-CP	0.159	0.159	0.160	0.165	0.169	0.166
				STE-PoS	0.116	0.117	0.120	0.132	0.142	0.136
82		0.9		STE-CP	0.144	0.145	0.147	0.150	0.153	0.151
				STE-PoS	0.093	0.094	0.097	0.107	0.115	0.110
83	4	0.4	H0	STE-CP	0.868	0.875	0.884	0.889	0.890	0.889
				STE-PoS	0.837	0.841	0.848	0.876	0.897	0.885
84		0.5		STE-CP	0.725	0.733	0.741	0.748	0.751	0.749
				STE-PoS	0.682	0.687	0.695	0.729	0.763	0.742
85		0.6		STE-CP	0.565	0.571	0.577	0.582	0.589	0.584
				STE-PoS	0.513	0.518	0.525	0.556	0.596	0.570
86		0.7		STE-CP	0.412	0.415	0.419	0.424	0.431	0.426
				STE-PoS	0.350	0.354	0.360	0.386	0.422	0.398
87		0.8		STE-CP	0.274	0.276	0.278	0.281	0.285	0.283
				STE-PoS	0.208	0.211	0.216	0.239	0.264	0.248
88		0.9		STE-CP	0.138	0.139	0.140	0.142	0.142	0.142
				STE-PoS	0.087	0.088	0.091	0.104	0.116	0.108
89	4	0.6	H1	STE-CP	0.294	0.301	0.311	0.317	0.321	0.317
				STE-PoS	0.247	0.252	0.260	0.295	0.334	0.310
90		0.7		STE-CP	0.179	0.182	0.185	0.188	0.194	0.190
				STE-PoS	0.141	0.144	0.148	0.166	0.193	0.175
91		0.8		STE-CP	0.109	0.110	0.111	0.113	0.116	0.114
				STE-PoS	0.074	0.075	0.078	0.089	0.104	0.094
92		0.9		STE-CP	0.055	0.056	0.056	0.057	0.057	0.057
				STE-PoS	0.029	0.030	0.031	0.037	0.042	0.039

Table S5: Probability of early termination (PET) for scenario 2 with 2 and 4 patients per month under various cutoff-calibrations.

simulation setting ID	patients per month	true p_1	hypothesis	design	Probability of early termination					
					Cutoff calibrated under short-term rate p_1					
					0.4	0.5	0.6	0.7	0.8	0.9
93	0.5	0.7	H0	STE-CP	-	-	-	0.743	0.741	0.744
				STE-PoS	-	-	-	0.759	0.756	0.762
94		0.75		STE-CP	-	-	-	0.741	0.738	0.742
				STE-PoS	-	-	-	0.756	0.752	0.759
95		0.8		STE-CP	-	-	-	0.740	0.738	0.742
				STE-PoS	-	-	-	0.752	0.748	0.755
96		0.85		STE-CP	-	-	-	0.737	0.735	0.739
				STE-PoS	-	-	-	0.744	0.741	0.747
97		0.9		STE-CP	-	-	-	0.738	0.735	0.740
				STE-PoS	-	-	-	0.738	0.735	0.741
98	0.5	0.85	H1	STE-CP	-	-	-	0.055	0.054	0.055
				STE-PoS	-	-	-	0.062	0.060	0.063
99		0.9		STE-CP	-	-	-	0.063	0.062	0.064
				STE-PoS	-	-	-	0.067	0.066	0.069
100	1	0.7	H0	STE-CP	-	-	-	0.728	0.726	0.729
				STE-PoS	-	-	-	0.769	0.765	0.772
101		0.75		STE-CP	-	-	-	0.720	0.717	0.721
				STE-PoS	-	-	-	0.755	0.752	0.758
102		0.8		STE-CP	-	-	-	0.714	0.711	0.716
				STE-PoS	-	-	-	0.739	0.736	0.742
103		0.85		STE-CP	-	-	-	0.708	0.706	0.710
				STE-PoS	-	-	-	0.721	0.718	0.724
104		0.9		STE-CP	-	-	-	0.699	0.697	0.701
				STE-PoS	-	-	-	0.700	0.697	0.704
105	1	0.85	H1	STE-CP	-	-	-	0.065	0.065	0.066
				STE-PoS	-	-	-	0.080	0.078	0.082
106		0.9		STE-CP	-	-	-	0.081	0.080	0.081
				STE-PoS	-	-	-	0.085	0.083	0.086
107	2	0.7	H0	STE-CP	-	-	-	0.711	0.710	0.712
				STE-PoS	-	-	-	0.808	0.803	0.811
108		0.75		STE-CP	-	-	-	0.663	0.661	0.663
				STE-PoS	-	-	-	0.765	0.760	0.768
109		0.8		STE-CP	-	-	-	0.612	0.611	0.613
				STE-PoS	-	-	-	0.712	0.707	0.717
110		0.85		STE-CP	-	-	-	0.568	0.566	0.570
				STE-PoS	-	-	-	0.654	0.648	0.658
111		0.9		STE-CP	-	-	-	0.519	0.518	0.521
				STE-PoS	-	-	-	0.586	0.581	0.591
112	2	0.85	H1	STE-CP	-	-	-	0.097	0.096	0.097
				STE-PoS	-	-	-	0.162	0.157	0.166
113		0.9		STE-CP	-	-	-	0.102	0.101	0.102
				STE-PoS	-	-	-	0.140	0.136	0.143
114	4	0.7	H0	STE-CP	-	-	-	0.664	0.664	0.664
				STE-PoS	-	-	-	0.900	0.895	0.904
115		0.75		STE-CP	-	-	-	0.566	0.566	0.567
				STE-PoS	-	-	-	0.838	0.831	0.843
116		0.8		STE-CP	-	-	-	0.468	0.466	0.470
				STE-PoS	-	-	-	0.753	0.745	0.759
117		0.85		STE-CP	-	-	-	0.372	0.370	0.376
				STE-PoS	-	-	-	0.638	0.628	0.645
118		0.9		STE-CP	-	-	-	0.271	0.266	0.275
				STE-PoS	-	-	-	0.493	0.484	0.502
119	4	0.85	H1	STE-CP	-	-	-	0.105	0.105	0.106
				STE-PoS	-	-	-	0.368	0.357	0.376
120		0.9		STE-CP	-	-	-	0.081	0.079	0.082
				STE-PoS	-	-	-	0.252	0.244	0.260

Table S6: Probability of early termination (PET) for scenario 3 with 0.5, 1, 2 and 4 patient(s) per month under various cutoff-calibrations.

simulation setting ID	patients per month	true p_1	hypothesis	design	Probability to reject H0: Cutoff calibrated under short-term rate p_1 0.4	0.5	0.6	0.7	0.8	0.9
1	0.5	0.3	H0	STE-CP	0.104	0.104	0.103	0.103	0.103	0.102
				STE-PoS	0.095	0.095	0.093	0.093	0.090	0.088
2		0.4		STE-CP	0.103	0.102	0.102	0.102	0.101	0.100
				STE-PoS	0.099	0.098	0.096	0.096	0.093	0.091
3		0.5		STE-CP	0.101	0.100	0.099	0.099	0.098	0.097
				STE-PoS	0.100	0.099	0.097	0.097	0.094	0.092
4		0.6		STE-CP	0.102	0.102	0.100	0.100	0.099	0.097
				STE-PoS	0.102	0.101	0.099	0.099	0.096	0.094
5		0.7		STE-CP	0.100	0.099	0.098	0.098	0.096	0.095
				STE-PoS	0.102	0.101	0.099	0.099	0.096	0.093
6		0.8		STE-CP	0.102	0.102	0.100	0.100	0.098	0.097
				STE-PoS	0.105	0.104	0.102	0.102	0.099	0.097
7		0.9		STE-CP	0.102	0.102	0.100	0.100	0.098	0.097
				STE-PoS	0.107	0.106	0.104	0.104	0.101	0.099
8	0.5	0.4	H1	STE-CP	0.840	0.840	0.839	0.839	0.838	0.836
				STE-PoS	0.813	0.810	0.804	0.804	0.795	0.789
9		0.5		STE-CP	0.824	0.823	0.821	0.821	0.819	0.817
				STE-PoS	0.814	0.812	0.808	0.808	0.800	0.795
10		0.6		STE-CP	0.820	0.819	0.817	0.817	0.813	0.810
				STE-PoS	0.820	0.818	0.813	0.813	0.804	0.799
11		0.7		STE-CP	0.813	0.811	0.808	0.808	0.804	0.801
				STE-PoS	0.818	0.816	0.810	0.810	0.801	0.796
12		0.8		STE-CP	0.809	0.808	0.805	0.805	0.800	0.797
				STE-PoS	0.821	0.818	0.812	0.812	0.803	0.798
13		0.9		STE-CP	0.804	0.803	0.799	0.799	0.793	0.790
				STE-PoS	0.819	0.817	0.812	0.812	0.804	0.797
14	1	0.3	H0	STE-CP	0.103	0.102	0.102	0.102	0.101	0.100
				STE-PoS	0.091	0.090	0.087	0.087	0.084	0.082
15		0.4		STE-CP	0.101	0.101	0.100	0.100	0.099	0.098
				STE-PoS	0.096	0.095	0.093	0.093	0.090	0.087
16		0.5		STE-CP	0.100	0.100	0.099	0.099	0.098	0.097
				STE-PoS	0.098	0.098	0.095	0.095	0.092	0.089
17		0.6		STE-CP	0.101	0.101	0.100	0.100	0.098	0.097
				STE-PoS	0.101	0.100	0.098	0.098	0.095	0.093
18		0.7		STE-CP	0.100	0.100	0.099	0.099	0.097	0.096
				STE-PoS	0.103	0.102	0.100	0.100	0.097	0.095
19		0.8		STE-CP	0.099	0.099	0.098	0.098	0.097	0.096
				STE-PoS	0.105	0.104	0.102	0.102	0.098	0.096
20		0.9		STE-CP	0.100	0.100	0.099	0.099	0.097	0.096
				STE-PoS	0.109	0.108	0.106	0.106	0.102	0.101
21	1	0.4	H1	STE-CP	0.821	0.820	0.819	0.819	0.816	0.813
				STE-PoS	0.780	0.778	0.771	0.771	0.759	0.752
22		0.5		STE-CP	0.798	0.797	0.796	0.796	0.792	0.789
				STE-PoS	0.787	0.785	0.779	0.779	0.769	0.762
23		0.6		STE-CP	0.787	0.786	0.783	0.783	0.778	0.775
				STE-PoS	0.789	0.787	0.780	0.780	0.770	0.763
24		0.7		STE-CP	0.776	0.775	0.772	0.772	0.767	0.764
				STE-PoS	0.789	0.787	0.781	0.781	0.770	0.763
25		0.8		STE-CP	0.768	0.766	0.763	0.763	0.758	0.755
				STE-PoS	0.789	0.787	0.780	0.780	0.769	0.763
26		0.9		STE-CP	0.762	0.761	0.758	0.758	0.753	0.750
				STE-PoS	0.793	0.790	0.784	0.784	0.773	0.767

Table S7: Type I error rate and power for scenario 1 with 0.5 and 1 patient(s) per month under various cutoff-calibrations.

simulation setting ID	patients per month	true p_1	hypothesis	design	Probability to reject H0					
					Cutoff calibrated under short-term rate p_1					
					0.4	0.5	0.6	0.7	0.8	0.9
27	2	0.3	H0	STE-CP	0.080	0.080	0.079	0.079	0.076	0.074
				STE-PoS	0.077	0.076	0.072	0.072	0.068	0.065
28		0.4		STE-CP	0.091	0.090	0.089	0.089	0.087	0.086
				STE-PoS	0.093	0.091	0.088	0.088	0.084	0.081
29		0.5		STE-CP	0.099	0.098	0.097	0.097	0.095	0.094
				STE-PoS	0.105	0.103	0.100	0.100	0.095	0.092
30		0.6		STE-CP	0.106	0.105	0.104	0.104	0.102	0.101
				STE-PoS	0.114	0.113	0.110	0.110	0.106	0.103
31		0.7		STE-CP	0.113	0.113	0.111	0.111	0.110	0.109
				STE-PoS	0.123	0.122	0.120	0.120	0.116	0.113
32		0.8		STE-CP	0.121	0.120	0.119	0.119	0.118	0.117
				STE-PoS	0.133	0.132	0.130	0.130	0.126	0.124
33		0.9		STE-CP	0.131	0.131	0.130	0.130	0.129	0.128
				STE-PoS	0.144	0.143	0.140	0.140	0.137	0.135
34	2	0.4	H1	STE-CP	0.696	0.695	0.692	0.692	0.676	0.661
				STE-PoS	0.682	0.676	0.661	0.661	0.643	0.631
35		0.5		STE-CP	0.716	0.715	0.710	0.710	0.702	0.696
				STE-PoS	0.733	0.728	0.716	0.716	0.699	0.689
36		0.6		STE-CP	0.737	0.735	0.730	0.730	0.722	0.719
				STE-PoS	0.767	0.763	0.753	0.753	0.737	0.727
37		0.7		STE-CP	0.755	0.753	0.749	0.749	0.744	0.741
				STE-PoS	0.792	0.789	0.781	0.781	0.767	0.758
38		0.8		STE-CP	0.771	0.770	0.767	0.767	0.763	0.760
				STE-PoS	0.813	0.811	0.803	0.803	0.791	0.784
39		0.9		STE-CP	0.792	0.791	0.789	0.789	0.785	0.783
				STE-PoS	0.838	0.835	0.828	0.828	0.818	0.812
40	4	0.3	H0	STE-CP	0.050	0.050	0.049	0.049	0.047	0.046
				STE-PoS	0.059	0.056	0.051	0.051	0.046	0.042
41		0.4		STE-CP	0.080	0.079	0.076	0.076	0.072	0.071
				STE-PoS	0.093	0.090	0.085	0.085	0.078	0.072
42		0.5		STE-CP	0.105	0.104	0.101	0.101	0.097	0.096
				STE-PoS	0.121	0.119	0.114	0.114	0.107	0.101
43		0.6		STE-CP	0.126	0.125	0.122	0.122	0.120	0.119
				STE-PoS	0.142	0.140	0.136	0.136	0.130	0.126
44		0.7		STE-CP	0.148	0.147	0.146	0.146	0.145	0.144
				STE-PoS	0.163	0.162	0.159	0.159	0.155	0.151
45		0.8		STE-CP	0.167	0.167	0.166	0.166	0.165	0.165
				STE-PoS	0.176	0.176	0.174	0.174	0.171	0.169
46		0.9		STE-CP	0.188	0.187	0.187	0.187	0.186	0.186
				STE-PoS	0.192	0.192	0.191	0.191	0.190	0.189
47	4	0.4	H1	STE-CP	0.488	0.486	0.478	0.478	0.465	0.461
				STE-PoS	0.530	0.518	0.491	0.491	0.461	0.437
48		0.5		STE-CP	0.634	0.630	0.616	0.616	0.597	0.592
				STE-PoS	0.687	0.677	0.655	0.655	0.625	0.599
49		0.6		STE-CP	0.738	0.733	0.721	0.721	0.706	0.702
				STE-PoS	0.794	0.787	0.772	0.772	0.749	0.727
50		0.7		STE-CP	0.815	0.813	0.808	0.808	0.802	0.799
				STE-PoS	0.865	0.862	0.851	0.851	0.835	0.821
51		0.8		STE-CP	0.884	0.884	0.882	0.882	0.880	0.877
				STE-PoS	0.916	0.914	0.908	0.908	0.900	0.892
52		0.9		STE-CP	0.936	0.935	0.934	0.934	0.933	0.931
				STE-PoS	0.950	0.949	0.946	0.946	0.942	0.939

Table S8: Type I error rate and power for scenario 1 with 2 and 4 patient per month under various cutoff-calibrations.

simulation setting ID	patients per month	true p_1	hypothesis	design	Probability to reject H0					
					Cutoff calibrated under short-term rate p_1					
					0.4	0.5	0.6	0.7	0.8	0.9
53	0.5	0.4	H0	STE-CP	0.102	0.102	0.101	0.099	0.098	0.099
				STE-PoS	0.099	0.098	0.097	0.093	0.090	0.091
54		0.5		STE-CP	0.099	0.099	0.098	0.096	0.096	0.096
				STE-PoS	0.099	0.099	0.098	0.095	0.093	0.094
55		0.6		STE-CP	0.098	0.098	0.097	0.096	0.095	0.095
				STE-PoS	0.100	0.100	0.099	0.096	0.095	0.096
56		0.7		STE-CP	0.099	0.099	0.098	0.097	0.095	0.096
				STE-PoS	0.103	0.102	0.102	0.099	0.097	0.098
57		0.8		STE-CP	0.100	0.099	0.099	0.097	0.096	0.096
				STE-PoS	0.105	0.104	0.104	0.101	0.099	0.100
58		0.9		STE-CP	0.097	0.097	0.096	0.094	0.092	0.093
				STE-PoS	0.103	0.103	0.102	0.100	0.097	0.099
59	0.5	0.6	H1	STE-CP	0.897	0.896	0.895	0.890	0.888	0.889
				STE-PoS	0.891	0.890	0.888	0.878	0.872	0.875
60		0.7		STE-CP	0.884	0.883	0.882	0.877	0.875	0.876
				STE-PoS	0.889	0.887	0.886	0.879	0.874	0.877
61		0.8		STE-CP	0.875	0.875	0.874	0.869	0.866	0.868
				STE-PoS	0.886	0.886	0.884	0.878	0.873	0.876
62		0.9		STE-CP	0.871	0.870	0.869	0.864	0.860	0.862
				STE-PoS	0.888	0.887	0.886	0.879	0.873	0.877
63	1	0.4	H0	STE-CP	0.098	0.097	0.097	0.095	0.095	0.095
				STE-PoS	0.091	0.090	0.089	0.086	0.084	0.085
64		0.5		STE-CP	0.097	0.097	0.097	0.094	0.094	0.094
				STE-PoS	0.096	0.096	0.095	0.092	0.089	0.091
65		0.6		STE-CP	0.098	0.097	0.097	0.095	0.094	0.095
				STE-PoS	0.100	0.099	0.099	0.096	0.093	0.095
66		0.7		STE-CP	0.095	0.095	0.095	0.093	0.092	0.093
				STE-PoS	0.100	0.100	0.099	0.096	0.094	0.095
67		0.8		STE-CP	0.095	0.095	0.094	0.093	0.091	0.092
				STE-PoS	0.103	0.102	0.101	0.098	0.096	0.098
68		0.9		STE-CP	0.094	0.094	0.093	0.092	0.091	0.091
				STE-PoS	0.104	0.104	0.103	0.100	0.098	0.099
69	1	0.6	H1	STE-CP	0.878	0.877	0.876	0.872	0.870	0.872
				STE-PoS	0.869	0.867	0.865	0.856	0.849	0.853
70		0.7		STE-CP	0.861	0.860	0.859	0.854	0.851	0.853
				STE-PoS	0.870	0.869	0.868	0.860	0.853	0.857
71		0.8		STE-CP	0.845	0.844	0.843	0.838	0.834	0.837
				STE-PoS	0.870	0.869	0.866	0.857	0.850	0.854
72		0.9		STE-CP	0.833	0.832	0.831	0.826	0.822	0.824
				STE-PoS	0.870	0.869	0.866	0.858	0.851	0.855

Table S9: Type I error rate and power for scenario 2 with 0.5 and 1 patient(s) per month under various cutoff-calibrations.

simulation setting ID	patients per month	true p_1	hypothesis	design	Probability to reject H0					
					Cutoff calibrated under short-term rate p_1					
					0.4	0.5	0.6	0.7	0.8	0.9
73	2	0.4	H0	STE-CP	0.082	0.082	0.081	0.080	0.079	0.080
				STE-PoS	0.081	0.080	0.079	0.074	0.070	0.072
74		0.5		STE-CP	0.088	0.087	0.087	0.085	0.084	0.085
				STE-PoS	0.091	0.090	0.089	0.084	0.081	0.083
75		0.6		STE-CP	0.095	0.094	0.094	0.093	0.092	0.092
				STE-PoS	0.100	0.100	0.099	0.095	0.092	0.094
76		0.7		STE-CP	0.100	0.100	0.100	0.098	0.097	0.098
				STE-PoS	0.108	0.108	0.107	0.103	0.100	0.102
77		0.8		STE-CP	0.107	0.107	0.107	0.106	0.105	0.106
				STE-PoS	0.118	0.117	0.116	0.113	0.111	0.112
78		0.9		STE-CP	0.112	0.112	0.112	0.111	0.110	0.111
				STE-PoS	0.124	0.124	0.123	0.121	0.119	0.120
79	2	0.6	H1	STE-CP	0.807	0.804	0.801	0.797	0.794	0.796
				STE-PoS	0.819	0.816	0.812	0.793	0.777	0.787
80		0.7		STE-CP	0.814	0.813	0.812	0.807	0.803	0.806
				STE-PoS	0.846	0.844	0.841	0.826	0.814	0.821
81		0.8		STE-CP	0.828	0.827	0.826	0.822	0.818	0.821
				STE-PoS	0.869	0.868	0.865	0.854	0.844	0.850
82		0.9		STE-CP	0.841	0.840	0.839	0.835	0.833	0.834
				STE-PoS	0.890	0.888	0.886	0.876	0.869	0.873
83	4	0.4	H0	STE-CP	0.055	0.054	0.051	0.050	0.050	0.050
				STE-PoS	0.066	0.064	0.062	0.055	0.047	0.052
84		0.5		STE-CP	0.083	0.081	0.080	0.079	0.078	0.078
				STE-PoS	0.092	0.091	0.089	0.083	0.076	0.080
85		0.6		STE-CP	0.103	0.102	0.101	0.101	0.099	0.100
				STE-PoS	0.112	0.111	0.110	0.106	0.100	0.104
86		0.7		STE-CP	0.116	0.116	0.116	0.115	0.114	0.115
				STE-PoS	0.125	0.125	0.124	0.121	0.116	0.119
87		0.8		STE-CP	0.129	0.128	0.128	0.128	0.128	0.128
				STE-PoS	0.136	0.136	0.136	0.133	0.131	0.132
88		0.9		STE-CP	0.140	0.140	0.140	0.139	0.139	0.139
				STE-PoS	0.145	0.145	0.145	0.144	0.143	0.143
89	4	0.6	H1	STE-CP	0.701	0.693	0.685	0.679	0.675	0.679
				STE-PoS	0.747	0.742	0.734	0.700	0.661	0.685
90		0.7		STE-CP	0.809	0.806	0.804	0.800	0.795	0.799
				STE-PoS	0.845	0.842	0.839	0.822	0.795	0.813
91		0.8		STE-CP	0.874	0.873	0.872	0.870	0.868	0.869
				STE-PoS	0.907	0.906	0.904	0.893	0.878	0.888
92		0.9		STE-CP	0.923	0.923	0.923	0.922	0.922	0.922
				STE-PoS	0.948	0.948	0.947	0.941	0.936	0.939

Table S10: Type I error rate and power for scenario 2 with 2 and 4 patients per month under various cutoff-calibrations.

simulation setting ID	patients per month	true p_1	hypothesis	design	Probability to reject H0					
					Cutoff calibrated under short-term rate p_1					
					0.4	0.5	0.6	0.7	0.8	0.9
93	0.5	0.7	H0	STE-CP	-	-	-	0.102	0.103	0.102
				STE-PoS	-	-	-	0.099	0.100	0.098
94		0.75		STE-CP	-	-	-	0.101	0.101	0.101
				STE-PoS	-	-	-	0.098	0.099	0.098
95		0.8		STE-CP	-	-	-	0.100	0.100	0.100
				STE-PoS	-	-	-	0.098	0.099	0.098
96		0.85		STE-CP	-	-	-	0.100	0.101	0.100
				STE-PoS	-	-	-	0.100	0.100	0.099
97		0.9		STE-CP	-	-	-	0.099	0.100	0.099
				STE-PoS	-	-	-	0.099	0.100	0.099
98	0.5	0.85	H1	STE-CP	-	-	-	0.935	0.935	0.935
				STE-PoS	-	-	-	0.929	0.930	0.928
99		0.9		STE-CP	-	-	-	0.926	0.927	0.925
				STE-PoS	-	-	-	0.922	0.923	0.921
100	1	0.7	H0	STE-CP	-	-	-	0.100	0.101	0.100
				STE-PoS	-	-	-	0.094	0.094	0.093
101		0.75		STE-CP	-	-	-	0.099	0.099	0.098
				STE-PoS	-	-	-	0.094	0.094	0.093
102		0.8		STE-CP	-	-	-	0.098	0.099	0.098
				STE-PoS	-	-	-	0.096	0.097	0.096
103		0.85		STE-CP	-	-	-	0.095	0.095	0.094
				STE-PoS	-	-	-	0.094	0.094	0.093
104		0.9		STE-CP	-	-	-	0.094	0.095	0.094
				STE-PoS	-	-	-	0.096	0.097	0.095
105	1	0.85	H1	STE-CP	-	-	-	0.924	0.924	0.923
				STE-PoS	-	-	-	0.911	0.913	0.909
106		0.9		STE-CP	-	-	-	0.908	0.909	0.907
				STE-PoS	-	-	-	0.905	0.907	0.904
107	2	0.7	H0	STE-CP	-	-	-	0.095	0.095	0.095
				STE-PoS	-	-	-	0.076	0.077	0.075
108		0.75		STE-CP	-	-	-	0.097	0.097	0.097
				STE-PoS	-	-	-	0.082	0.083	0.081
109		0.8		STE-CP	-	-	-	0.100	0.101	0.100
				STE-PoS	-	-	-	0.088	0.089	0.087
110		0.85		STE-CP	-	-	-	0.101	0.101	0.101
				STE-PoS	-	-	-	0.093	0.093	0.092
111		0.9		STE-CP	-	-	-	0.102	0.102	0.102
				STE-PoS	-	-	-	0.097	0.098	0.096
112	2	0.85	H1	STE-CP	-	-	-	0.893	0.893	0.892
				STE-PoS	-	-	-	0.831	0.835	0.828
113		0.9		STE-CP	-	-	-	0.885	0.886	0.885
				STE-PoS	-	-	-	0.851	0.854	0.847
114	4	0.7	H0	STE-CP	-	-	-	0.098	0.098	0.098
				STE-PoS	-	-	-	0.046	0.047	0.044
115		0.75		STE-CP	-	-	-	0.103	0.103	0.102
				STE-PoS	-	-	-	0.057	0.059	0.056
116		0.8		STE-CP	-	-	-	0.107	0.107	0.106
				STE-PoS	-	-	-	0.070	0.071	0.069
117		0.85		STE-CP	-	-	-	0.115	0.115	0.114
				STE-PoS	-	-	-	0.085	0.086	0.084
118		0.9		STE-CP	-	-	-	0.120	0.121	0.120
				STE-PoS	-	-	-	0.099	0.100	0.098
119	4	0.85	H1	STE-CP	-	-	-	0.884	0.884	0.883
				STE-PoS	-	-	-	0.629	0.639	0.621
120		0.9		STE-CP	-	-	-	0.903	0.904	0.901
				STE-PoS	-	-	-	0.740	0.747	0.732

Table S11: Type I error rate and power for scenario 3 with 0.5, 1, 2 and 4 patients per month under various cutoff-calibrations.
